# Bacteria Synergized with PD‐1 Blockade Enhance Positive Feedback Loop of Cancer Cells‐M1 Macrophages‐T Cells in Glioma

**DOI:** 10.1002/advs.202308124

**Published:** 2024-03-23

**Authors:** Qi Chen, Yuyi Zheng, Xiaojie Chen, Yuan Xing, Jiajie Zhang, Xinyi Yan, Qi Zhang, Di Wu, Zhong Chen

**Affiliations:** ^1^ Interdisciplinary Institute for Medical Engineering Fuzhou University Fuzhou Fujian 350108 China; ^2^ Key Laboratory of Neuropharmacology and Translational Medicine of Zhejiang Province School of Pharmaceutical Sciences Zhejiang Chinese Medical University Hangzhou Zhejiang 310053 China

**Keywords:** anti‐PD‐1, cancer immunotherapy, customized bacteria, glioma, tumor microenvironment

## Abstract

Cancer immunotherapy is an attractive strategy because it stimulates immune cells to target malignant cells by regulating the intrinsic activity of the immune system. However, due to lacking many immunologic markers, it remains difficult to treat glioma, a representative “cold” tumor. Herein, to wake the “hot” tumor immunity of glioma, *Porphyromonas gingivalis* (Pg) is customized with a coating to create an immunogenic tumor microenvironment and further prove the effect in combination with the immune checkpoint agent anti‐PD‐1, exhibiting elevated therapeutic efficacy. This is accomplished not by enhancing the delivery of PD‐1 blockade to enhance the effect of immunotherapy, but by introducing bacterial photothermal therapy to promote greater involvement of M1 cells in the immune response. After reaching glioma, the bacteria further target glioma cells and M2 phenotype macrophages selectively, enabling precise photothermal conversion for lysing tumor cells and M2 phenotype macrophages, which thereby enhances the positive feedback loop of cancer cells‐M1 macrophages‐T cells. Collectively, the bacteria synergized with PD‐1 blockade strategy may be the key to overcoming the immunosuppressive glioma microenvironment and improving the outcome of immunotherapy toward glioma.

## Graphical abstract

*Porphyromonas gingivalis* (Pg) synergized with PD‐1 blockade strategy is reported. After reaching glioma, the customized bacteria can target glioma cells and M2 phenotype macrophages selectively, receive and convert light into heat for lysing tumor cells and M2 phenotype macrophages, thereby enhancing the positive feedback loop of cancer cells‐M1 macrophages‐T cells.

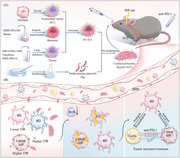

## 1 Introduction

Glioma is a devastating tumor of the central nervous system.^[^ ^1^, ^2^ ^]^ Unfortunately, due to the blurred boundaries, high heterogeneity, and aggressiveness of brain gliomas, conventional treatments including surgery, chemotherapy, and radiotherapy have limited therapeutic effects,^[^ ^3^, ^4^ ^]^ giving results of poor prognosis, short survival, more metastases, and high recurrence rate.^[^ ^5^, ^6^ ^]^ As a pillar of cancer therapy, cancer immunotherapy is expected to control and eliminate glioma cells utilizing the body's immune system.^[^ ^7^ ^]^ Among treatment patterns for cancer immunotherapy, immune checkpoint therapy anti‐PD‐1 specific to co‐stimulatory molecule PD‐1 which is a member of the B7‐CD28 superfamily has yielded encouraging results in fighting against many types of cancers, especially in hematological tumors.^[^ ^8^ ^]^ However, the phase III clinical trials of anti‐PD‐1 showed low therapeutic effects in glioma and failed to benefit glioma patients.^[^ ^9^ ^]^ Therefore, it remains urgent to develop a new and effective strategy for improving the outcome of anti‐PD‐1 in glioma treatment.

The following challenges have been found in anti‐PD‐1 immunotherapy in glioma: 1) The lack of tumor antigens within the brain is central to the failure of stimulating immune cells against glioma cells.^[^ ^10^ ^]^ 2) The existence of the blood‐brain barrier (BBB), which is composed of continuous brain endothelial cells, astrocytes, microglia, and pericytes,^[^ ^11^ ^]^ not only protects the brain from viruses and toxins in the blood circulation but also prevents biopharmaceuticals such as anti‐PD‐1 from entering the brain. 3) Over‐production of reactive oxygen species (ROS) in the brain provides stressful living conditions for immune cells.^[^ ^12^ ^]^ These challenges have guided us to develop strategies to further increase anti‐PD‐1 efficacy for durable clinical responses.

The total number of bacteria on the epidermis and in the body is about ten times the number of human cells.^[^ ^13^ ^]^ Due to their unique advantages such as a wide range of sources, and small individuals, bacteria have been used in bioimaging, diagnosis, and treatment.^[^ ^14^ ^]^ The exploitation of bacteria in tumor therapy dates back to the 19th century.^[^ ^15^ ^]^ Numerous studies showed that anaerobic bacteria could use flagella to swim into the deep oxygen‐deprived tumor microenvironment and act as foreign antigens to increase the immunogenicity of tumors.^[^ ^16^ ^]^ Inspired by previous research by Stephen S. Dominy et al., *Porphyromonas gingivalis* (Pg) was identified in the brain of Alzheimer's disease patients,^[^ ^17^ ^]^ we noticed Pg may potentially be utilized in the development of treatments for brain diseases. Moreover, our previous findings corroborated Pg's ability to secrete melanin and it can be used as a whole for photothermal therapy for subcutaneous tumors.^[^ ^18^ ^]^ However, the environment of glioma is more complex than that of subcutaneous tumors. How to modify Pg is the central question to apply Pg to glioma in the brain. Recent developments of engineered bacteria which overexpressed respiratory chain enzyme II by Zhang et al. and engineered bacteria which modified nucleic acid aptamers on the surface by Liu et al. highlighted the applications of synthetic biology methods.^[^ ^19^, ^20^ ^]^ But the yield and preparation time remains difficult to control. In this regard, we have fabricated customized bacteria based on chemical and physical methods.

**Figure 1:**
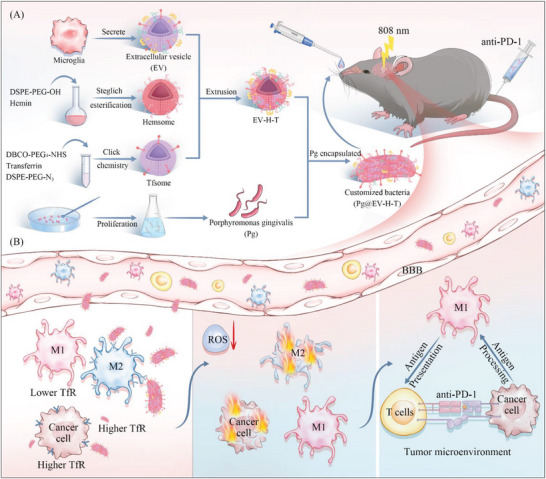
Schematic illustration of bacteria synergized with PD‐1 blockade to enhance the positive feedback loop of cancer cells‐M1 macrophages‐T cells in glioma. A) The process of constructing bacterial coating EV‐H‐T. B) The functional pattern of the customized bacteria. Customized bacteria cross the BBB, target higher TfR expressed glioma cells and M2 macrophages resulting in selective death under the laser, and relieve the survival stress of lived M1 macrophages, which further improves the effect of anti‐PD‐1 via the enhanced cross‐talks among cancer cells, M1 macrophages, and T cells.

Here, we introduce synergistic therapy to increase tumor antigens, improve the permeability of BBB or bypass the BBB, as well as to regulate ROS levels to relieve environmental stress for immune cell survival. In this design, customized bacteria were administrated via the nasal cavity for the on‐demand awakening of the “hot” tumor immunity of glioma (**Figure** **1**A,B). Pg was selected as the main module for photothermal therapy to lyse tumor cells and generate supplementary tumor antigens. As Pg is being applied to glioma for the first time, modifications for biocompatibility improvement should be conducted for glioma treatment. It has been reported that coating bacteria with biocompatible lipid membranes can afford microbiota with multi‐function.^[^ ^21^, ^22^ ^]^ Although some studies found that there is a correlation among Pg, periodontal disease, and brain health, for example, patients with generalized periodontitis often have comorbidities and are considered at risk of developing Alzheimer's disease,^[^ ^23^ ^]^ encapsulated Pg cannot recognize the surrounding environment, which affects the interaction between Pg and brain cells, lowering the risk.^[^ ^24^ ^]^ To realize the abilities of the customized bacteria, we designed a bacterial coating consisting of extracellular vesicles (EV) from microglia and synthetic liposomes (Hemsome and Tfsome). EV, natives in the glioma microenvironment, can easily cross the BBB and be home to microglia.^[^ ^25^ ^]^ Tfsome is synthesized by click chemistry for active targeting. Transferrin (Tf) can specifically bind to the Tf receptor (TfR). As a glycoprotein, the TfR has two subunits of 90 kDa connected by a disulfide bond and participates in the transcytosis of cellular iron, and each subunit can bind to one molecule of Tf.^[^ ^26^ ^]^ TfR‐mediated transcytosis is considered an effective approach to drug transportation across the BBB.^[^ ^27^ ^]^ Interestingly, in addition to glioma cells, M2 immunosuppressive macrophages also have higher TfR expression levels in comparison to M1 macrophages.^[^ ^28^ ^]^ To the best of our knowledge, it is the first time that Tf‐modified systems were found to cross the BBB as well as selectively bind to M2 macrophages and glioma cells. Additionally, with the rapid development of immunotherapy and nanotechnology, several studies have reported regulation of M1/M2 phenotype could serve as a strategy to reprogram macrophages for enhanced photothermal‐immunotherapy.^[^ ^29^, ^30^ ^]^ However, it remains uncovered that how M1/M2 ratio changes is affected by macrophage repolarization and/or M2‐type macrophage apoptosis. This inspired us to functionalize the bacteria with Tfsome for selective targeting and photothermal inhibition of M2 macrophages. We also provided a clear explanation for the increase in the M1/M2 ratio in our strategy, which contributes to higher immune activity, as a result of the reduction in M2 macrophages. Hemsome is synthesized by Steglich esterification for introducing hemin into our customized bacteria. Hemin, an iron‐binding porphyrin, can play a catalytic role in decomposing ROS produced by tumor cell metabolism. Because ROS can cause lipid peroxidation and adversely affect immune cells, the integration of hemin onto bacteria helps to adsorb ROS in the tumor microenvironment, easing the survival stress of M1 cells after laser treatment.

As we know, the activation of naive T cells requires at least two of the following three signals, which are antigen signals expressed on the surface of antigen‐presenting cells, co‐stimulatory molecule B7‐CD28, and cytokine IL‐12.^[^ ^31^, ^32^ ^]^ To improve the efficacy of anti‐PD‐1 which acts on B7‐CD28, our strategy is to make more M1 cells alive, ensuring the processes of antigen processing and antigen presentation. What's more, previous research showed that macrophages appear to be beneficial for the survival of patients with glioblastoma.^[^ ^33^ ^]^ Modulating macrophages to complement traditional immune checkpoint therapy could be an effective way.

In addition, intranasal administration, as a non‐invasive route, can achieve rapid delivery of bacteria to brain.^[^ ^34^ ^]^ Efficiencies of brain‐targeted delivery via different administration routes were compared and it was found that intranasal administration is an optimal method for delivering our customized bacteria.

Together, taking advantage of the intrinsic photothermal effect of bacteria, the extrinsic selective targeting ability of EV and Tfsome, and the extrinsic ROS relieving behavior of Hemsome, the customized bacteria are demonstrated to wake the “hot” tumor immunity and boost the anti‐PD‐1 immunotherapy in glioma through the enhancement of the positive feedback loop of cancer cells‐M1 macrophages‐T cells.

## 2 Results and Discussion

### 2.1 Developing the Customized Bacteria

**Figure 2:**
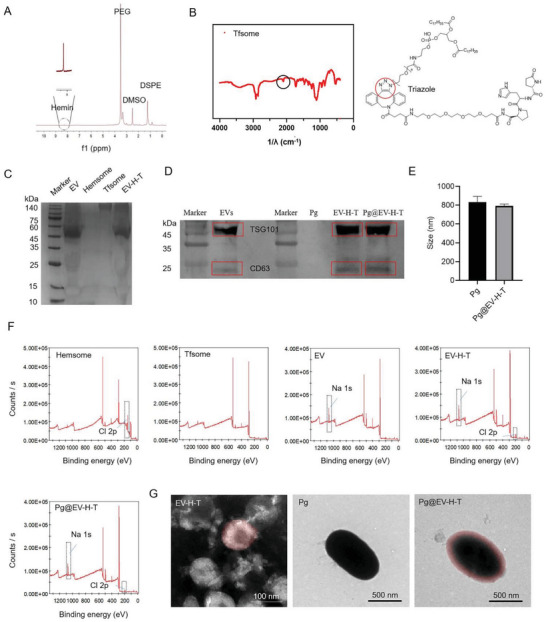
Characterizations of the customized bacteria. A) ^1^H‐NMR spectra of Hemsome in DMSO‐d6. B) FT‐IR spectra of Tfsome. The circle indicates the structure of the triazole. C) SDS‐PAGE of EV, Hemsome, Tfsome, and EV‐H‐T. D) WB of EVs, Pg, EV‐H‐T and Pg@EV‐H‐T. E) Size of Pg and Pg@EV‐H‐T. Data presented as mean ± SD, *n =* 6. F) A wide energy range XPS spectrum was recorded from a thin film sample of Hemsome, Tfsome, EV, EV‐H‐T, and Pg@EV‐H‐T, respectively. G) Representative TEM images of the EV‐H‐T, Pg, and Pg@EV‐H‐T.

The customized bacteria are composed of four parts, EV, Hemsome, Tfsome, and Pg, each of which carries out the respective function. We first cultivated primary microglia and studied how the cell membrane (CM) or EV from the BV2 microglial cells interacted with them (Figure S1, Supporting Information). The results revealed EV induces more phenotypic changes of primary microglia than CM although they both stimulate the polarization of microglia. While the proportion of M1 and M2 cells increased by CM was approximate, the M2 cells increased by EV was more than M1 cells which illustrates EV may be more suitable as our starting material. Moreover, in the cytokine test, the CM group was more inclined to produce cytokines secreted by M1 while the EV group was more inclined to produce cytokines secreted by M2, which agrees with the above findings. Based on the pre‐experimental evidence, EV was chosen for our customized bacteria. Then we synthesized Hemsome by coupling the carboxyl group of hemin and the hydroxyl group of DSPE‐PEG‐OH (Figure S2A, Supporting Information). Successful synthesis was confirmed by the hydrogen‐1 NMR (^1^H‐NMR) (**Figure** **2**A) and liquid chromatography‐mass spectrometry (LC‐MS) (Figure S3, Supporting Information) spectra. Tf was conjugated to DBCO‐PEG_4_‐NHS, followed by the azide‐alkyne click reaction with DSPE‐PEG‐N_3_ to yield Tfsome (Figure S2B,C, Supporting Information). Fourier‐transform infrared spectroscopy (FT‐IR) showed the feature of triazole near 2000 cm^−1^ which was also circled out in the chemical structural formula of Tfsome (Figure 2B) and sodium dodecyl sulfate‐polyacrylamide gel electrophoresis (SDS‐PAGE) with PEG‐color reaction image demonstrated both Tf and PEG were located in Tfsome (Figure S4, Supporting Information). Then, we prepared the bacterial coating EV‐Hemsome‐Tfsome (EV‐H‐T) by mixing and co‐extruding the solutions of EV, Hemsome, and Tfsome. The resulting EV‐H‐T had protein bands from EV and Tfsome (Figure 2C). Particle diameter and polydispersity index (PDI) of each component of EV‐H‐T were analyzed (EV, ≈100 nm; Hemsome, ≈250 nm; Tfsome, ≈150 nm; PDI under 0.6; Figure S5, Supporting Information). We developed the customized bacteria (Pg@EV‐H‐T) by coating EV‐H‐T onto the surface of Pg and detected exosomal markers TSG101 and CD63 which were still maintained in the final customized bacteria using western blot (WB) (Figure 2D and Figure S6, Supporting Information). We quantified the content of Pg and EV‐H‐T in Pg@EV‐H‐T using Turbidimetric determination at 670 nm and BCA protein concentration determination at 562 nm respectively (Figure S7, Supporting Information). The average absorbance in both wavelengths was similar indicating the amount of free Pg and free EV‐H‐T were the same as that of Pg and EV‐H‐T in Pg@ EV‐H‐T. The size of customized bacteria was similar to Pg, barely affected by the coating (Figure 2E and Figure S8, Supporting Information). The obtained customized bacteria were also characterized by X‐ray photoelectron spectroscopy (XPS) to mainly show ‐Cl groups from the hemsome present on the surface, which supplement what was not proved by the above protein immunoblot experiments about the exist of hemsome in the whole (Figure 2F). The vesicle shape of EV‐H‐T was irregular after negative staining and the rod shape of the bacteria remained after coating (Figure 2G; Figures S9 and S10, Supporting Information). As shown in Figure S11 (Supporting Information), the customized bacteria were further observed using confocal laser scanning microscopy. Pg and EV‐H‐T were labeled with 4′,6‐diamidino‐2‐phenylindole (DAPI), and fluorescein isothiocyanate (FITC), respectively. Pg displayed blue fluorescence, and EV‐H‐T displayed green fluorescence. The customized bacteria exhibited overlapped fluorescence of green and blue, which indicated the successful coating of Pg by EV‐H‐T.

The ability of the bacteria to produce melanin is essential for later photothermal combined research. Thus, we compared the bacterial growth (Figure S12, Supporting Information) and the production of melanin (Figure S13, Supporting Information) before and after the coating. The coating had limited impact on affect the growth of bacteria and the production of melanin which were promoted by the presence of nutrient hemin. The customized bacteria can also darken the culture medium visually (Figure S14, Supporting Information). All these results demonstrated that the customized bacteria were obtained as expected and can be further conducted for the functional pattern.

### 2.2 Functions of the Customized Bacteria In Vitro

**Figure 3:**
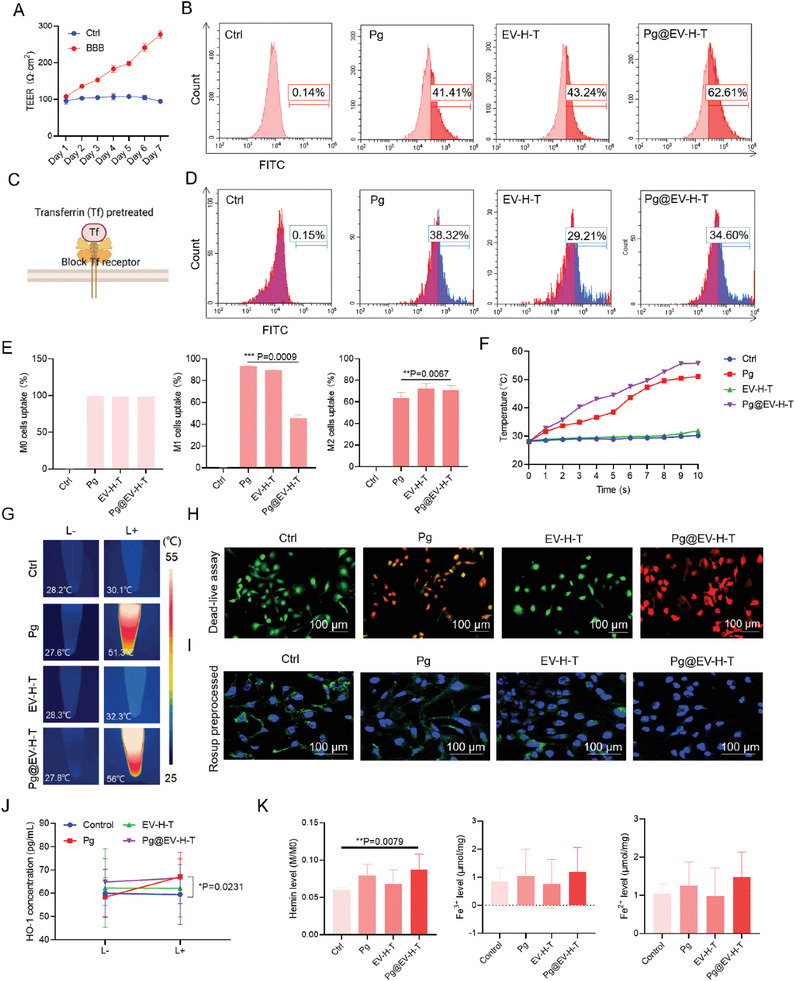
The functional pattern of the bacteria in vitro. A) The transepithelial electrical resistance (TEER) was measured to prove the construction of the BBB model. Data presented as mean ± SD, *n =* 6. B) The penetration rates of Pg, EV‐H‐T, and Pg@EV‐H‐T. C) Schematic diagram of Tf receptor blocked model for evaluating whether the customized bacteria could use the Tf‐TfR transport pathway. D) The penetration rates of Pg, EV‐H‐T, and Pg@EV‐H‐T after the block of TfR. E) Preferential uptake by macrophages with different phenotypes. Data presented as mean ± SD, *n =* 6, *p<0.05, **p<0.01, *** *p<*0.001. F) Temperature rising curve of Pg, EV‐H‐T and Pg@EV‐H‐T. G) Infrared thermal imaging of Pg, EV‐H‐T, and Pg@EV‐H‐T with or without 808 nm laser irradiation. L‐ indicated without laser. L+ indicated with laser. H) Calcein‐AM/PI staining images of glioma cells treated with Pg, EV‐H‐T or Pg@EV‐H‐T and irradiated with 808‐nm laser (0.33 W cm^−2^, 10 min). Scale bar = 100 µm. I) Confocal fluorescent images of ROS levels in BV2 cells with different treatments and irradiated with 808‐nm laser (0.33 W cm^−2^, 10 min). The cells were stained by green fluorescence DCFH‐DA and blue fluorescence Hoechst. Scale bar = 100 µm. J) Heme oxygenase 1 (HO‐1) expression in BV2 cells. L‐ indicated without laser. L+ indicated with laser. Data presented as mean ± SD, *n =* 6, *p<0.05, **p<0.01, *** *p<*0.001. K) The hemin, Fe^3+^, and Fe^2+^ levels in BV2 cells incubated with PBS, Pg, EV‐H‐T, or Pg@EV‐H‐T. Data presented as mean ± SD, *n =* 6, *p<0.05, **p<0.01, *** *p<*0.001.

The customized bacteria were tested for the capability of BBB‐permeability. To investigate the BBB crossing, a co‐cultured transwell model with bEnd.3 cells in the upper chamber and glioma cells in the lower chamber were built (Figure S15, Supporting Information). We checked the values of transepithelial electrical resistance (TEER) for seven days and began cell studies when the values exceeded 200 Ω cm^−2^ (**Figure** **3**A). Fluorescein isothiocyanate (FITC) was labeled on Pg, EV‐H‐T, and Pg@EV‐H‐T respectively. Compared with Pg and EV‐H‐T groups, the glioma cells at the bottom treated with Pg@EV‐H‐T showed more cells with green fluorescence, which implied that the EV‐H‐T modification can assist Pg to cross the BBB (Figure 3B). Next, to investigate the role of Tf in the system, cells were pre‐processed with 100 µg mL^−1^ Tf solution for 0.5 h (Figure 3C). To answer whether the uptake of Pg, EV‐H‐T, and Pg@EV‐H‐T is related to Tf concentration and incubation time, we used different Tf concentrations (25, 50, and 100 µg mL^−1^) and various incubation times (0.5, 1 and 2 h) to treat cells (Figure S16, Supporting Information). The results showed that the incubation Tf concentration increases above 100 µg mL^−1^ can inhibit the uptake, especially the EV‐H‐T and Pg@EV‐H‐T group. Inhibition can be achieved after only 0.5 h of coculture with Tf. Our findings agreed with the literature that the process of Tf binding to its receptor is dynamic.^[^ ^35^ ^]^ Prolonged incubation time will affect the effectiveness. We found that Pg, EV‐H‐T, and Pg@EV‐H‐T showed similar percentages of fluorescence cells because EV‐H‐T and Pg@EV‐H‐T were affected by blocked TfR (Figure 3D). As we know, besides glioma cells, it is also necessary to focus on the uptake of different phenotypes of macrophages for our customized bacteria which aim at selective targeting of M2 phenotypes. Thus, lipopolysaccharides (LPS) or IL‐4 were used as stimuli to trigger the differentiation of resting macrophage (M0 phenotype) to M1 or M2 phenotypes.^[^ ^36^ ^]^ Firstly, we examined the expression of TfR on macrophages with different phenotypes (Figure S17, Supporting Information). M0, M1, and M2 macrophages were collected and lysed. Equal amounts of protein from M0, M1, and M2 macrophage groups were loaded and probed with an anti‐CD71 (Transferrin Receptor) primary antibody that recognizes the target protein of interest. M2 macrophages showed the highest expression of TfR, M0 macrophages displayed a moderate level, while M1 macrophages exhibited the lowest expression of TfR. Thereafter, we used M0, M1, and M2 macrophages to assess the cellular uptake of Pg, EV‐H‐T, and Pg@EV‐H‐T by flow cytometry after labeling with FITC (Figure 3E). The results showed no significant variance of Pg, EV‐H‐T, and Pg@EV‐H‐T in M0 cells. However for M1 cell uptake, when compared to the Pg group, Pg@EV‐H‐T showed a significant decrease. At the same time, there was a significant increase of Pg@EV‐H‐T in M2 cell uptake in comparison with Pg, indicating that the bacterial coating was acting in favor of the M2 target.

When customized bacteria were located in target cells, the next step was to evaluate the photothermal effect on the target area. Therefore, in vitro, we studied the photothermal properties of the sample solution (Figure 3F,G). The laser power we used was 0.33 W cm^−2^ according to the safety limit for the 808 nm laser in brain tumors.^[^ ^37^ ^]^ The temperature of Pg and Pg@EV‐H‐T solutions reached nearly 50°C in 10 minutes. Such temperature could result in glioma cell death (Figure 3H). It could be seen from the results that because Pg can absorb light and convert it into heat, it causes indiscriminate damage to macrophages. After engineering into Pg@EV‐H‐T, Pg@EV‐H‐T had an obvious M2 phenotype tendency, so most dead cells were M2 phenotype (Figure S18, Supporting Information). In addition to dead/live staining, we also investigated the heat‐induced toxicity of components of customized bacteria by Cell Counting Kit‐8 (CCK‐8) assay (Figure S19, Supporting Information). After exposure to EV‐H‐T, no significant decrease in cell viability was observed, whereas a dose‐dependent decrease in cell viability was observed after exposure to Pg or Pg@EV‐H‐T at ranges of 2.5 × 10^6^ CFU to 12.5 × 10^6^ CFU.

Hemsome immobilized on the surface of the customized bacteria could work as ROS nanoscavengers against ROS‐induced immune cell damage. First, we quantified hemin in the customized bacteria by the standard curve of hemin (Figure S20, Supporting Information). We incubated BV2 cells with Rosup agents which leads to ROS generation prior to the treatment. Then, the ROS‐scavenging activity was measured by dichlorodihydrofluorescein diacetate (DCFH‐DA) fluorescence (Figure 3I; Figures S21 and S22, Supporting Information). Hemsome, EV‐H‐T, and Pg@EV‐H‐T showed diminished green fluorescent intensity coming from DCFH‐DA, indicating ROS‐scavenging activity. We also used free radical scavenging experiments to test the antioxidant capacity of our customized bacteria (Figure S23, Supporting Information). After mixing radical probes with Pg@EV‐H‐T, the color of solutions changed from dark to light, accompanied by a decrease in the absorption curve. Besides, we assessed M1 macrophage function and viability in the presence of induced ROS (Figures S24 and S25, Supporting Information). The result showed that the fluorescence intensity of the DCFH‐DA probe in M1 cells treated with Pg@EV‐H‐T was optimally attenuated, furthermore, with laser, Pg@EV‐H‐T could reduce more ROS. We then assessed macrophage function including pinocytosis, antigen presentation function, and the secretion of immune‐related cytokines, as well as the viability. The results demonstrated that the treatment of Pg@EV‐H‐T would not cause the death of M1 cells. After treatment, the pinocytosis function of macrophages was enhanced. M1 cell surface proteins used for antigen presentation function were not lost. The ability of M1 cells to secrete cytokines still existed.

Because heme oxygenase 1 (HO‐1) has anti‐oxidative properties and responses to oxidative stressors,^[^ ^38^ ^]^ we also evaluated the HO‐1 expression in BV2 cells before and after laser treatment (Figure 3J). The Pg@EV‐H‐T displayed the highest HO‐1 level among all treatments, demonstrating it protected the immune cells from ROS damage. In addition, the hemin, Fe^3+^, and Fe^2+^ levels in BV2 cells were slightly elevated, which emphasized the ROS depletion response involved by Hemsome on Pg@EV‐H‐T (Figure 3K). There was no significant difference in Fe^3+^ and Fe^2+^ levels indicating negligible ferroptosis of BV2 cells.

Furthermore, we used a transwell system to test whether T cells can be stimulated by macrophages ex vivo (Figure S26, Supporting Information). We used Rosup to stimulate M1 cells in the lower layer first, and after treatment with Pg, EV‐H‐T, and Pg@EV‐H‐T, spread T cells on the upper layer to examine the proliferation of T cells (CFSE staining) and the expression of CD86 in M1 cells. As the results showed, M1 cells from the Pg@EV‐H‐T treated group enhanced T cell proliferation and the expression of CD86 significantly.

### 2.3 Evaluations of Customized Bacteria In Vivo

**Figure 4:**
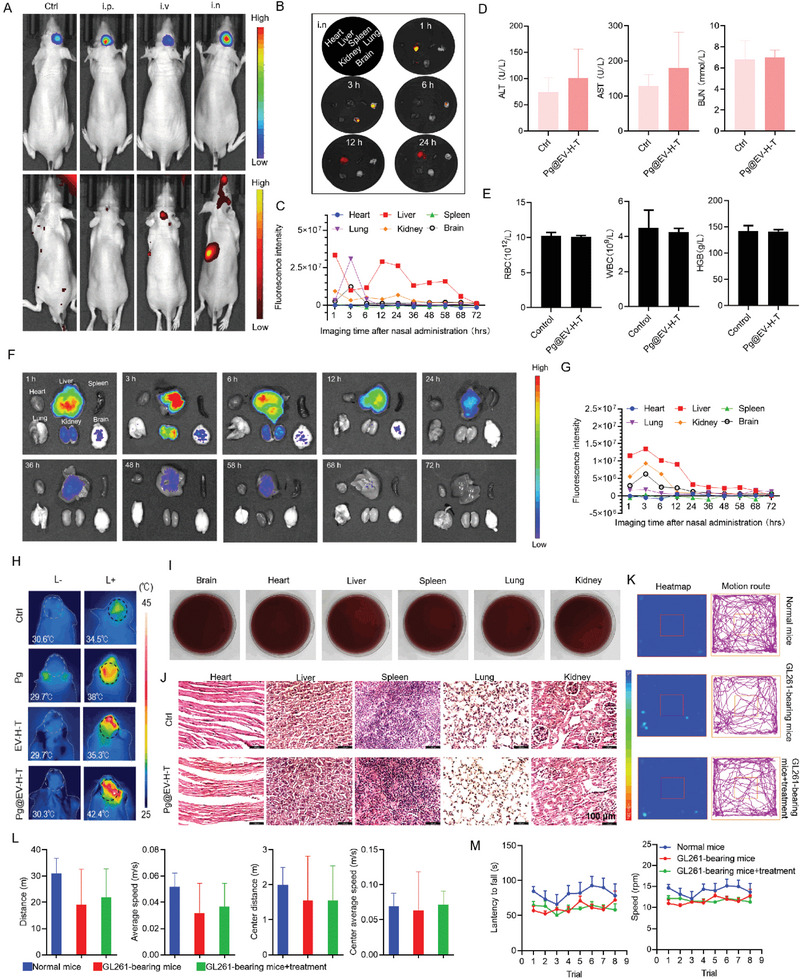
Targeting, photothermal performance, and biocompatibility of the bacteria in vivo. A) Luciferase luminescence images and fluorescence images of mice bearing U87‐Luc tumors after Pg@EV‐H‐T treatments through different routes. i.p., intraperitoneal injection; i.v., intravenous injection; i.n., intranasal administration. B) Ex vivo fluorescence imaging of major tissues of U87‐bearing mice at 1, 3, 6, 12, and 24 h post intranasal administration of DiR‐labeled customized bacteria. C) Quantitative analysis of the fluorescent signals of main tissues to show the decomposition of Pg@EV‐H‐T in U87‐bearing mice. D) ALT, AST, and BUN test at 3 h after Pg@EV‐H‐T administration to study liver and kidney function. Data presented as mean ± SD, *n =* 6. E) RBC, WBC, and HGB tests at 3 h after Pg@EV‐H‐T administration to study whether customized bacteria influence the blood system. Data presented as mean ± SD, *n =* 6. F) Ex vivo fluorescence imaging of major tissues of GL261‐bearing mice at different time points post intranasal administration of DiR‐labeled customized bacteria. G) Quantitative analysis of the fluorescent signals of main tissues in F. H) Infrared thermal images of treatment groups. L‐indicated without laser. L+ indicated with laser. I) After laser treatment, homogenates of the main organs were collected and coated on the blood agar. J) H&E staining of heart, liver, spleen, lung, and kidney after the treatment of Pg@EV‐H‐T. Scale bar = 100 µm. K) Athletic analysis of groups of normal mice, glioma‐bearing mice, and glioma‐bearing mice with Pg@EV‐H‐T treatment. Motion route and heatmap of each group in the open‐field test. L) Distance, average speed, center distance, and center average speed of the test mice. Data presented as mean ± SD, *n =* 6. M) Rotarod test results of different groups, including time on rotarods and speed. Data presented as mean ± SD, *n =* 6.

To further figure out whether the customized bacteria can play the designed roles in vivo and have good biocompatibility, which provides important guidance to the next anti‐glioma experiments, we conducted a series of corresponding studies in glioma models. We examined different routes for Pg@EV‐H‐T administration first. As shown in **Figure** **4**A, we compared intraperitoneal injection (i.p.), intravenous injection (i.v.), and intranasal administration (i.n.) in firefly luciferase stably transfected U87 (U87‐Luc) tumors. After 3 h post‐administration, fluorescence signals from DiR‐labeled customized bacteria could be found in groups of all these three routes, however, for i.p. and i.v., most bacteria had been metabolized. Hence, i.n. was chosen for our customized bacteria administration. Owing to epithelial cells may be the major obstacle for i.n.,^[^ ^39^ ^]^ we used the human bronchial epithelial cell line (16‐HBE) to observe the intercept of Pg@E‐H‐T (Figure S27, Supporting Information). Pg and Pg@EV‐H‐T have scales in microns, which led to an easier block than nano EV‐H‐T.

Next, at different time points, we extracted major organs from mice treated with customized bacteria through i.n. (Figure 4B). Ex vivo organ distribution results indicated that Pg@EV‐H‐T can easily get into the liver, brain, and lung. At 3 h post‐administration, the brain had obvious fluorescence which validated the in vivo results. In view of the enrichment of Pg@EV‐H‐T in the liver and kidney, we further conducted the decomposition of Pg@EV‐H‐T in the liver and kidney in U87‐bearing mice (Figure 4C and Figure S28, Supporting Information). At 36 h, the fluorescence of Pg@EV‐H‐T in the kidney was no longer obvious, and the metabolism of Pg@EV‐H‐T in the liver was completed by 72 hours.

Then, blood biochemistry and blood routine examinations were used to study the effects of customized bacteria on liver and kidney functions, as well as the blood system at 3 h after Pg@EV‐H‐T administration. This timing was chosen because the brain exhibited the highest accumulation of Pg@EV‐H‐T at that point and it was speculated that Pg@EV‐H‐T could be consumed in the brain after performing photothermal operations (Figure 4D,E). There was no obvious alteration, in alanine aminotransferase (ALT), aspartate aminotransferase (AST), urea nitrogen (BUN), red blood cells (RBC), white blood cells (WBC), and hemoglobin (HGB) of Pg@EV‐H‐T group, as compared to that of the control group.

In addition, we detected the accumulation and distribution of Pg@EV‐H‐T in GL261‐bearing mice (Figure 4F,G). The degradation rate in the brain of GL261‐bearing mice was slower than in nude mice. Both U87‐bearing mice and GL261‐bearing mice exhibited gradual degradation of Pg@EV‐H‐T.

Regarding hemolysis being a fundamental issue for safe administration in vivo, we evaluated Pg and Pg@EV‐H‐T by incubating them with red blood cells (Figure S29, Supporting Information). Up to 1 × 10^7^ CFU of customized bacteria had no potential risk of hemolysis for each mouse during every administration.

We continued to validate that the parameters for photothermal are safe in mice. After i.n. of Pg, EV‐H‐T, and Pg@EV‐H‐T, the brains of mice were exposed to 0.33 W cm^−2^ 808 nm irradiation for 10 min. An infrared thermal camera was used to record thermal images at the initial irradiation stage and at the end of the irradiation stage. The temperature of the customized bacteria group increased to 42.4°C which realized mild photothermal therapy (Figure 4H). We assessed the potential for cyclic heating of Pg, EV‐H‐T, and Pg@EV‐H‐T (Figure S30, Supporting Information). After four laser on/off cycles, there was no obvious change in the temperature curves. After four sessions of laser treatment, the main organs were collected for making homogenized samples. Then, homogenized samples were spread on blood agar and cultured for three days to observe residual bacteria (Figure 4I). Bacteria did not proliferate further on the blood plates. Histomorphology of the heart, liver, spleen, lung, and kidney was also assessed through hematoxylin and eosin (H&E)‐stained images (Figure 4J). The H&E‐stained images showed customized bacteria group had no obvious abnormality.

The central nervous system is vulnerable to hyperthermia.^[^ ^40^ ^]^ To validate that the strategy of Pg@EV‐H‐T assisted photothermal therapy does not affect the central nervous system of mice, we tested the exercise capacities of mice related to the neuromuscular system (Figure 4K–M). Heatmap of travel pathway and motion route of illustrative examples of normal, GL261‐bearing mice and GL261‐bearing mice with Pg@EV‐H‐T treatment (GL261‐bearing mice + treatment) filmed in the open field test arena. Normal mice traveled a longer distance than glioma‐bearing mice. Surprisingly, mice from the group of GL261‐bearing mice + treatment entered the central area of the open field more frequently and faster than mice from the group of GL261‐bearing mice, although inferior to normal mice. Moreover, rotarod tests that could reflect the balance and coordination of mice were used to demonstrate that our treatment did not harm the central nervous system. GL261‐bearing mice group and GL261‐bearing mice + treatment group showed lower functional performance in the rotarod than normal mice, which indicated that the existence of glioma affected the neuromuscular system and our treatment failed to help.

### 2.4 Improving the Outcome of Anti‐PD‐1 in Glioma Treatment

**Figure 5:**
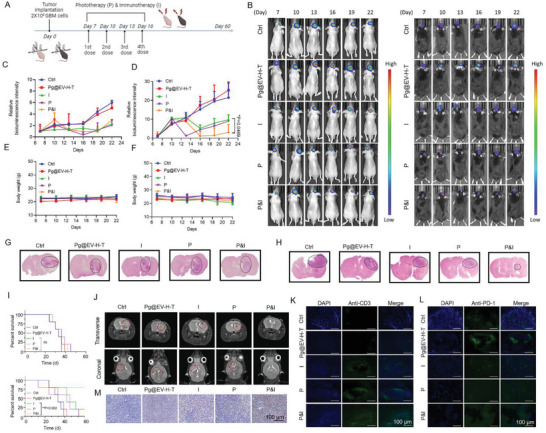
Anti‐glioma effects of bacteria synergized with PD‐1 blockade. A) Schematic illustrating the strategy of customized bacteria‐combined photothermal immunotherapy. I, immunotherapy; P, photothermal therapy; P&I, the combination of photothermal therapy and immunotherapy. B) Tumor growths of U87‐Luc or GL261‐Luc were monitored by bioluminescence imaging at days 7, 10, 13, 16, 19, and 22. C) The variance of relative bioluminescence intensity in U87‐bearing nude mice. Data presented as mean ± SD, *n =* 6. D) The variance of relative bioluminescence intensity in GL261‐bearing C57 mice. Data presented as mean ± SD, *n =* 6, *p<0.05, **p<0.01, *** *p<*0.001. E) The changes in body weight of the orthotopic U87‐bearing mice. Data presented as mean ± SD, *n =* 6. F) The changes in body weight of the orthotopic GL261‐bearing mice. Data presented as mean ± SD, *n =* 6. G) H&E staining images of the brains from orthotopic U87‐bearing mice with different treatments. The black circle pointed to the tumor region. H) H&E staining images of the brains from orthotopic GL261‐bearing mice with different treatments. The black circle pointed to the tumor region. I) The survival rate curves of the orthotopic U87‐bearing or GL261‐bearing mice in different treatment groups. *n =* 6, *p<0.05, **p<0.01, *** *p<*0.001. J) T2W‐MRI imaging of GL261‐bearing mice treated with Pg@EV‐H‐T, immunotherapy, phototherapy, and combined photothermal immunotherapy. The red circles indicated the location of the tumors. K) Immunofluorescence images for analyzing the expression of anti‐CD‐3 in different treatments in GL261 glioma models. Blue: cell nuclei. Green: anti‐CD‐3 staining. Scale bar = 100 µm. L) Immunofluorescence images for analyzing the expression of anti‐PD‐1 in different treatments in GL261 glioma models. Blue: cell nuclei. Green: anti‐PD‐1 staining. Scale bar = 100 µm. M) Immunohistochemistry of TUNEL in the brain slides from GL261 glioma models with different treatments.

To distinguish the effects of customized bacteria in improving the outcome of anti‐PD‐1, nude mice that lack thymus and C57BL/6 mice which has intact immune systems were selected for constructing glioma experimental models. The therapeutic schedule was presented in **Figure** **5**A, where anti‐PD‐1 was injected through the tail vein instantly after intranasal administration of customized bacteria. Three hours later, laser treatment was given to glioma experimental models. We recorded the tumor volume by monitoring the bioluminescence intensity from U87‐Luc cells or GL261‐Luc cells. U87‐Luc cells were provided by the company while GL261‐Luc cells were transfected by our lab (Figure S31, Supporting Information). As shown in Figure 5B, bioluminescence imaging of tumor growth of U87‐Luc or GL261‐Luc was monitored every three days. We compared the anti‐glioma efficacy of five groups: 1. Control (Ctrl) group; 2. Pg@EV‐H‐T group; 3.Immunotherapy (I) group which was treated with anti‐PD‐1; 4. Photothermal therapy (P) group which was treated with customized bacteria and laser; 5. Combination of photothermal therapy and immunotherapy (P&I) group which was treated with anti‐PD‐1, customized bacteria, and laser. The relative bioluminescence intensity indicated in nude mice, neither bacteria‐based photothermal therapy nor anti‐PD‐1 immunotherapy generates inconspicuous tumor inhibition (Figure 5C). This phenomenon could be mainly ascribed to the defective immune system and the lack of trans‐urocanic acid and melanin to absorb laser irradiation. When C57BL/6 mice were employed to perform the same operation, the P&I group presented a higher therapeutic outcome than the I group, showing synergistic effects with anti‐PD‐1 therapy (Figure 5D). Of importance, Pg@EV‐H‐T could not shrink tumors alone if it does not cooperate with anti‐PD‐1 and photothermal operations. In addition, slight body weight fluctuation was observed (Figure 5E,F).

Consistent with the results of bioluminescence imaging, H&E‐stained brain tissues of the glioma‐bearing mice showed no sign of significant tumor growth inhibition in nude mice but significant tumor growth inhibition in C57BL/6 mice, at 22 days after implantation, which indicates T cells involved in the immune response are essential for tumor suppression (Figure 5G,H). To be noted, the survival rate of orthotopic GL261‐glioma mice treated with P&I was above 80% until day 60 compared to only 20% treated with I (Figure 5I). Besides, we also used T2‐weighted magnetic resonance imaging (T2W‐MRI) to scan mice brains and found at day 22, I or P‐treated GL261‐bearing mice had a relatively smaller tumor size than those in ctrl group or in Pg@EV‐H‐T group (Figure 5J). Due to the resolution of T2W‐MRI, it was even difficult for us to detect the demarcation of tumor region in the P&I group, suggesting our customized bacteria exhibited enhanced performance.

At the end of the anti‐glioma experiment, the increased distribution of T cells in the tumor area of the P&I group was analyzed by anti‐CD3 and anti‐PD‐1 immunofluorescence (Figure 5K,L). Moreover, the glioma cell apoptosis was assessed by immunohistochemistry of caspase3, cleaved caspase3, and terminal deoxynucleotidyl transferase dUTP nick end labeling (TUNEL) (Figure 5M and Figure S32, Supporting Information). Nonconforming activation of caspase3 can lead to tumorigenesis instead of inducing programmed cell death.^[^ ^41^ ^]^ All groups showed abnormal expression of caspase3. The P&I group was relatively better. In contrast to that in control group, Pg@EV‐H‐T group and I group, the expression levels of cleaved caspase‐3 were significantly higher in P group and P&I group. Comparatively, the highest expression of apoptotic DNA fragmentation was observed in the P&I group. Meanwhile, we ascertained the histological analysis (Figure S33, Supporting Information). At the cellular level, there were no abnormalities illustrating all the treatments were safe.

### 2.5 Enhancing the Positive Feedback Loop of Cancer Cells‐Macrophages‐T Cells

**Figure 6:**
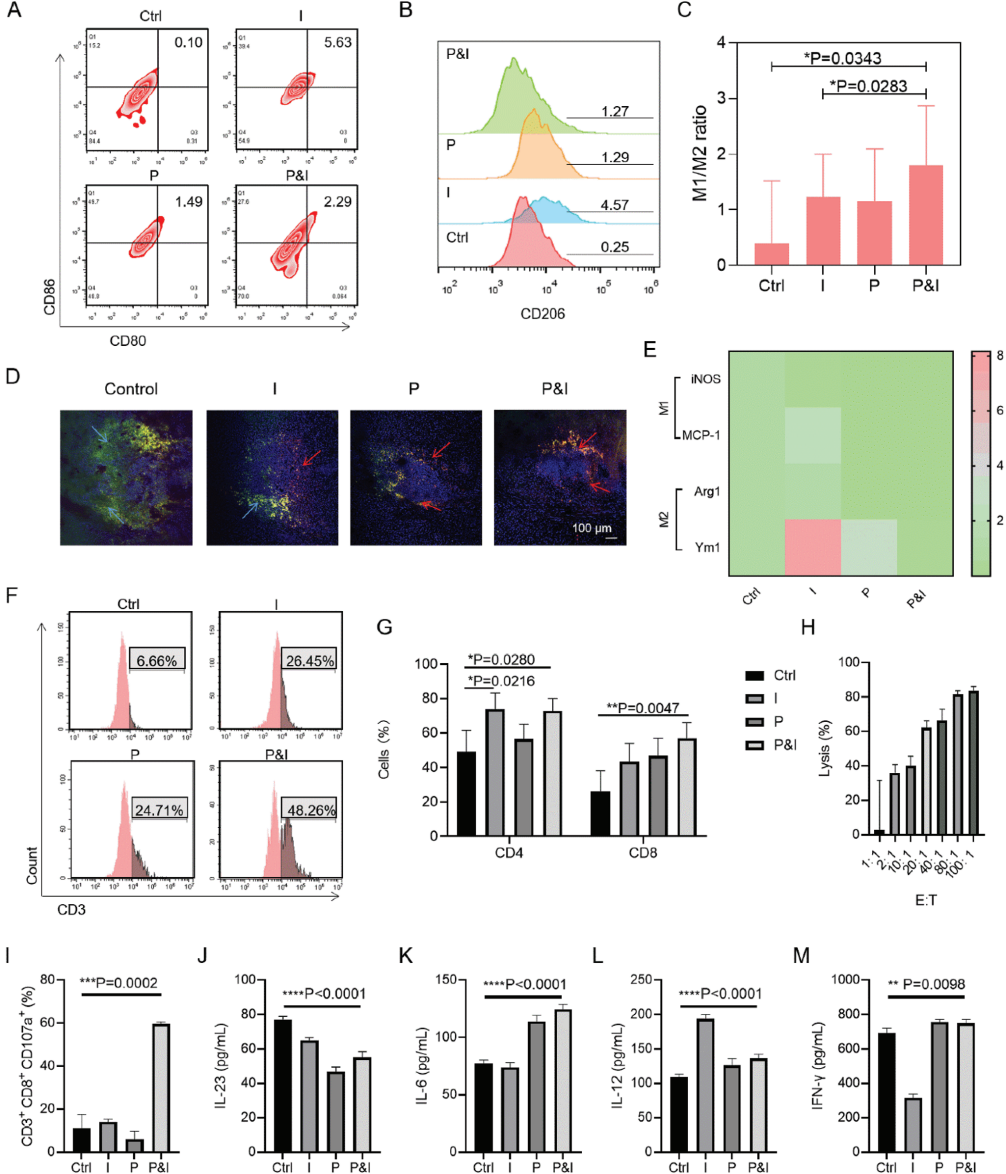
The analysis of the immune microenvironment induced by bacteria synergized with PD‐1 blockade. A) Flow cytometric analysis of M1 cells (CD80hi&CD86hi) in the brains after different treatments. B) Flow cytometric analysis of M2 cells (CD206hi) in the brains after different treatments. C) The M1/M2 ratio in the brains after different treatments. Data presented as mean ± SD, *n =* 6, *p<0.05, **p<0.01, *** *p<*0.001. D) Immunofluorescence of DAPI, PE‐CD86, and FITC‐CD206 on cryosectioned mice brain tissue. The green arrows represented the expression of CD206 and the red arrows pointed to the expression of CD86. E) Total RNA was gathered from the whole brain, and mRNA expression level of iNOS, MCP‐1, Arg1, and Ym1 was assayed through qPCR using GAPDH as an internal standard. F) Flow cytometry on brain cells to count CD3^+^ T cells population. G) Identification of CD4^+^ or CD8^+^ T cell population under the gate of CD3^+^ T cells. Data presented as mean ± SD, *n =* 6, *p<0.05, **p<0.01, *** *p<*0.001. H) The cytotoxicity measured at different effector:target (E:T) ratios. Effectors were T cells from P&I group. Target was glioma cells. Data presented as mean ± SD, *n =* 6, *p<0.05, **p<0.01, *** *p<*0.001. I) Percentages of CD3^+^ CD8^+^ CD107a^+^ cells. Data presented as mean ± SD, *n =* 6, *p<0.05, **p<0.01, *** *p<*0.001. J) Expression levels of IL‐23 were quantitatively tested by ELISA. Data presented as mean ± SD, *n =* 6, *p<0.05, **p<0.01, *** *p<*0.001. K) Expression levels of IL‐6 in the brain. Data presented as mean ± SD, *n =* 6, *p<0.05, **p<0.01, *** *p<*0.001. L) Expression levels of IL‐12 in the brain. Data presented as mean ± SD, *n =* 6, *p<0.05, **p<0.01, *** *p<*0.001. M) Expression levels of IFN‐γ in the brain. Data presented as mean ± SD, *n =* 6, *p<0.05, **p<0.01, *** *p<*0.001.

To further verify that our proposed customized bacteria can wake the “hot” tumor immunity of glioma, we collected brains from each group and made them into single cells for the tumor microenvironment analysis. Firstly, we analyzed the phenotype of tumor‐associated macrophages (TAMs). The dissociated cells were stained by APC‐labelled CD11b, FITC‐labelled CD206, PerCP‐Cyanine5.5‐labelled CD80, and PE‐labelled CD86. Although the proportion of M1 cells (CD80hi&CD86hi) and M2 cells (CD206hi) in tumors was increased after anti‐PD‐1 treatment, the M1/M2 ratio was decreased as compared to P&I groups (**Figure** **6**A–C). We intended to figure out why both immunotherapy (I) and photothermal therapy (P) alone can induce the increase of M1 and M2, but the effect of P&I combined was inferior to that of P or I alone. Immunotherapy used anti‐PD‐1 to treat mice so that it could induce the increase of M1 and M2. Photothermal therapy uses a laser to treat glioma so that it could increase the tumor lysates as autoantigens which further enhanced the antigen presentation involving M1 and M2 macrophages when compared to the control group and the meantime reduced the numbers of M1 and M2 cells when compared to I group. P&I showed a moderate level in M1 cells and a minimum M2 cell level when compared to the I or P group. We then evaluated M1/M2 ratio at earlier time points (Day 16 v. Day 19 v. Day 22) (Figure S34, Supporting Information). The ratio of M1/M2 showed a trend of increasing and then decreasing. Among all treatments, the P&I treatment showed the smallest decrease in the M1/M2 ratio. These results indicated that immunotherapy could increase the infiltration of macrophages, whereas photothermal therapy could decrease the infiltration of macrophages. Ultimately, owing to the mutual influence of immunotherapy and photothermal therapy, the P&I group, selectively reduced more M2 cells than M1 cells. We then used frozen sections to co‐stain markers for M1 cells (CD86) and M2 cells (CD206) (Figure 6D). The control group primarily showed the infiltration of suppressive M2 cells. The Pg@EV‐H‐T group increased the infiltration of M1 cells. The two phenotypes of macrophages in I group appeared to be proportionally balanced macroscopically, primarily focusing on the periphery of the tumor. P group reduced the number of M2 macrophages. Results in P&I group also demonstrated a similar distribution of macrophages as P group. We further found MCP‐1, Arg1, and Ym1 mRNA expression were downregulated, and maintained at moderate levels, which verified the capability of P&I to modulate the balance of M1 and M2 cells (Figure 6E).

We next sought to identify the activity of T cells which act as the downstream cells after the macrophages process antigen information from cancer cells and present on the surface. More T cells (CD3‐positive) had already been involved in antigen presentation in the P&I group, approaching 48.26%, almost twice that of the other groups, demonstrating a possible direct impact from the balance of M1 and M2 cells to T cells (Figure 6F). Increased T cell subtypes were also observed in mice exposed to I, P, and P&I (Figure 6G). Interestingly, P&I had the highest CD8^+^ amounts among all groups, suggesting remarkably increased anti‐glioma immune response as they played a prominent role in identifying and killing cancer cells. The obvious difference in CD4‐positive amounts between I and P&I were not shown. We cocultured T cells from P&I group (effector cells) and tumor cells (target cells) to measure the lysis of tumor cells (Figure 6H). As the proportion of effector cells increased, the killing effect on tumor cells increased. Up to 80% lysis effect was achieved. In addition, the expression of CD3^+^ CD8^+^ CD107a^+^, a biomarker of tumor‐specific cytotoxic T lymphocytes (CTLs), was evaluated (Figure 6I). P&I group presented up to 60% CD3^+^ CD8^+^ CD107a^+^ expression.

It was also important to determine the role of CD4^+^ cells in the glioma microenvironment. Thus, when checking the cytokines secreting by CD4^+^ cells, we found the level of IL‐4 in the P&I group was lower than the I group, and the level of IL‐1β in the I and P&I group were similar (Figure S35, Supporting Information). IL‐4 promotes T helper 2 (Th2) response which has been proposed to facilitate tumor growth, while IL‐1β expression induces robust and durable CD4 responses.^[^ ^42^, ^43^ ^]^ The expression of IL‐1β matched with the differentiation results of T cell subtypes. The expression of IL‐4 suggested P&I group assisted in the relief of the immunosuppressive glioma microenvironment through attenuated Th2 responses. We also tested more cytokines, including IL‐23, IL‐6, IL‐12, and IFN‐γ (Figure 6J–M). IL‐23 is an important factor in the differentiation and maintenance of Th17 cells, which is considered to be associated with the development and invasion of tumors, as they can mediate inflammatory responses and promote neovascularization.^[^ ^44^ ^]^ IL‐6 triggers apoptosis and inhibits in vivo growth of malignant T cells.^[^ ^45^ ^]^ IL‐12 has been reported to exert IFN‐γ‐independent tumor‐suppressive activities.^[^ ^46^ ^]^ IFN‐γ which could be produced by CD8^+^ cytotoxic T lymphocytes, has a key role in preventing tumor initiation, growth, and metastasis.^[^ ^46^ ^]^ The downregulation of IL‐23 expression and the upregulation of IL‐6, IL‐12, and IFN‐γ expression could be observed in P&I group when compared to saline‐treated group, demonstrating the antitumor immune response elicited by the treatment. Taken together, our study indicated that the positive feedback loop of cancer cells‐macrophages‐T cells existed under the bacteria‐based P&I strategy.

## 3 Conclusion

These customized bacteria are based on Pg, EV, Hemsome, and Tfsome, in combination with an 808 nm laser for improving the efficacy of anti‐PD‐1 toward glioma. Compared with other engineered bacteria, the formulations of our customized bacteria could face three challenges of anti‐PD‐1 immunotherapy for glioma, exerting its functions at a clear pattern (BBB passing→selective target→photothermal conversion→oxidative damage protection) with high safety profiles. We verified each step in the functional pattern. We pioneered intranasal administration of customized Pg for brain delivery and even M2 macrophage delivery. Upon intranasal administration, Pg@EV‐H‐T could bypass the BBB barrier. In case some of them cannot use the way around, the EV part and Tfsome part in Pg@EV‐H‐T can provide active homing to the glioma microenvironment. After arriving at the glioma, the Tfsome part in Pg@EV‐H‐T can target TfR‐overexpressed cancer cells and M2 cells that have more TfR, which enables the next step of photothermal conversion. Pg@EV‐H‐T in cancer cells and M2 cells rely on melanin to absorb heat from an 808 nm irradiation, producing leakage antigens as neoantigens. The part of Hemsome in Pg@EV‐H‐T can consume ROS via the reaction of ROS+Fe^3+^→Fe^2+^+H_2_O+O_2_, preventing live M1 cells from oxidative damage.

We have demonstrated that Pg@EV‐H‐T is biodegradable and has no obvious side effects. The mild temperature used in our strategy avoids potential thermal injuries to the fragile central nervous system. However, current data are not enough to draw conclusions that the fragments from damaged Pg@EV‐H‐T can be completely eliminated by bodies. Future studies will be required to focus on this question and answer whether fragments trigger foreign body reactions.

We have evaluated the anti‐glioma effects of anti‐PD‐1 immunotherapy, Pg@EV‐H‐T based photothermal therapy, and Pg@EV‐H‐T combined anti‐PD‐1 photothermal immunotherapy in U87‐bearing nude mice and GL261‐bearing C57BL/6 mice. The prerequisite to improve anti‐PD‐1 efficacy is having a complete immune system, especially the presence of T cells which are targets of anti‐PD‐1. Pg@EV‐H‐T‐based photothermal therapy worked as assistance to anti‐PD‐1 immunotherapy in the GL261 glioma model, ameliorating tumor volume and survival rate. The number of both PD‐1 expressed T cells and apoptotic cells increased after the treatment of Pg@EV‐H‐T combined anti‐PD‐1 photothermal immunotherapy, apparently waking the “hot” tumor immunity.

We have tested the hypothesis behind the appearance of a “hot” tumor that customized bacteria combined with anti‐PD‐1 photothermal immunotherapy can enhance the positive feedback loop of cancer cells‐macrophages‐T cells. We analyzed the molecules expressed on macrophages and T cells in glioma. A higher M1/M2 ratio in tumors could be seen in the photothermal immunotherapy group. The balance of M1/M2 cells supported antigen processing and antigen presentation, resulting in more cytotoxic T cells and helper T cells but with less IL‐4 expression. The observed positive correlation between the number of macrophages and T cells supported the hypothesis of a positive feedback loop.

In summary, this work disclosed the versatility of Pg@EV‐H‐T in completing the mission of selective targeting and waking the asleep immune system. Pg@EV‐H‐T‐based combination therapy could be extended to other cold tumors to improve the sensibilization of anti‐PD‐1, for instance, taking into account the specific characteristics of different tumor immune microenvironments and replacing the bacterial coating. We believe our customized bacteria will stimulate more innovative thinking on fabricating engineered bacteria and using them as novel systems for disease intervention and treatment.

## 4 Experimental Section

### Materials

Pg strain ATCC33277 was obtained from BeNa Culture Collection Center (Beijing, China). Anti‐CD63 &amp; anti‐TSG101 were bought from Umibio (Shanghai, China). Exosome‐depleted FBS was provided by HAKATA (Shanghai, China). Exo‐spin mini standard and exo‐spin buffer were bought from TheWell Bioscience (North Brunswick, USA). LPS, hemin, and transferrin were purchased from Sigma‐Aldrich (St. Louis, USA). Mouse IL‐4 was purchased from PeproTech (RockyHill, USA). Firefly luciferase reporter gene lentiviral vector was bought from Genechem (Shanghai, China). DiR and FITC were bought from Beyotime Biotechnology (Shanghai, China). DCFH‐DA and Rosup were bought from Solarbio (Beijing, China). HO‐1 Assay Kit, Ferrous Iron Colorimetric Assay Kit, and Cell Total Iron Colorimetric Assay Kit were provided by Elabscience (Wuhan, China). n situ Apoptosis Detection Kit was bought from TaKaRa (Beijing, China). APC‐CD11b, FITC‐CD206, Percp/Cy5.5‐CD80, PE‐CD86, FITC‐CD3, PE‐CD4, Percp/Cy5.5‐CD8, FITC‐PD1 was bought from BioLegend (San Diego, USA). Mouse IL‐4 and Mouse IL‐1 ELISA Kits were bought from Boster (Pleasanton, USA).

### Animals

Six to eight weeks male C57BL/6 and male BALB/c nude mice were provided by CharlesRiver Laboratories (Beijing, China) and cultivated in a specific pathogen‐free environment in accordance with China Animal Protection Law (CAPN). The animal protocols were approved by the Zhejiang Chinese Medical University Laboratory Animal Research Center Ethics Committee (approval number: IACUC‐20231023‐05) and relied on by all animal experiments.

### Cell Culture

The U87, GL261, BV2, and bEnd.3 cell lines were purchased from the American Type Culture Collection (ATCC). Primary microglia were extracted from fetal mice, followed by culture for 10 days in IL‐4 (20 ng mL^−1^) or LPS (100 ng mL^−1^) supplemented medium to obtain M2‐polarized cells or M1‐polarized cells.

### Preparation of Customized Bacteria

1) The preparation of EVs: BV2 cells were grown to 90% confluency, washed three times, and incubated with serum‐free media for another 48 h. The supernatant was collected and centrifuged at 300×g for 10 min to remove cells and cellular debris. Transferred the supernatant to a new tube and centrifugated at 16 000×g for 30 min to remove the remained cellular debris. The resulting supernatant was subsequently added with Exo‐spin precipitation buffer and incubated overnight. Precipitation of EV was obtained by centrifugation at 16 000×g for 1 h. Added 100 µL PBS to resuspend the EV for further purification. A size exclusion column was first put at room temperature for 15 min to equilibrate and 250 µL PBS was added to wet. Transferred the 100 µL EV into the size exclusion column and collected purified EV through gravity. 2) The preparation of Hemsome: 100 mg DSPE‐PEG‐OH, 16 mg Hemin, 4.58 mg DMAP, 23 mg EDC, 0.5 mL DMF, and 50 mL anhydrous dichloromethane were mixed and stirred for 48 h at room temperature without water, oxygen and light. The final product Hemsome was washed 3 times using anhydrous dichloromethane and dialyzed using MW2000 regenerated cellulose dialysis tape. 3) The preparation of Tfsome: 1 µg µL^−1^ DBCO‐PEG_4_‐NHS was sterilized using a 0.22 µm cell filter and added into 0.1 µg µL^−1^ transferrin solution. Then the solution was put on the cell shaker under 300 rpm for 1 h at room temperature. The solution was centrifuged at 7000 r min^−1^ for 30 min to remove the excess DBCO‐PEG_4_‐NHS using a 15 mL 30 KDa MerckMillipore ultrafiltration tube. The trapped solution was further mixed with DSPE‐PEG‐N_3_ at 37 °C for 1 h under 40 r min^−1^, dialyzed using MW3500 regenerated cellulose dialysis tape, and stored for the preparation of EV‐H‐T. 4) The preparation of EV‐H‐T: EV, Hemsome, and Tfsome were mixed together at the volume ratio of 1:1:1 at 37 °C for 1 h under 40 r min^−1^. 5) The preparation of Pg@EV‐H‐T: 1 mL 10^7^ CFU mL^−1^ Pg was resuspended in PBS, mixed with 100 µL EV‐H‐T and cultured at 37°C for 2 h. The mixture was extruded 11 times through a polycarbonate porous membrane, followed by centrifugation at 4000 rpm for 20 min to remove excess EV‐H‐T.

### Characterization of Customized Bacteria

Hemsome was assayed by ^1^H‐NMR spectra using DMSO‐d6 as the solvent and by HPLC‐MS spectrometry using water and acetonitrile as eluents. Tfsome was assayed by FT‐IR spectrum from 4000 to 400 cm^−1^ in the infrared region and by SDS‐PAGE using PEG color reaction. EV was assayed by SDS‐PAGE to detect the main protein stripes and by WB using CD63 and TSG101 as markers. Suspensions containing Pg, EV‐H‐T, and Pg@EV‐H‐T were visualized by TEM. The generation of Pg@EV‐H‐T was proved by XPS to study the changes in surface elements and by DLS to analyze the changes in particle size.

### Fabrication of In Vitro Blood‐Brain Barrier (BBB) Model and the Penetration Efficiency

A transwell system was used to fabricate the in vitro BBB model. bEnd.3 cells at the density of 5 × 10^4^ cells were seeded in the upper chamber. The lower chamber was placed blank medium. The transmembrane resistance of bEnd.3 cells were recorded every day using a transmembrane resistance meter. When the resistance reached and stabilized above 200 Ω cm^2^, it was observed under a microscope to ensure the construction of the simulated BBB in vitro. Cancer cells were added into the Transwell lower chamber one day in advance, and then FITC‐labeled EV, Hemsome, Tfsome, and EV‐H‐T were added the next day. After 3 h incubation, the cells in the lower chamber were collected, and the proportion of cells expressing fluorescence was recorded by flow cytometry to study the penetration efficiency of the BBB.

### The Selective Target to Higher Tf Receptor (TfR) Expressed Glioma Cells and M2 Phenotype Macrophages

To clarify the role of Tf in brain‐targeted transport, the cells in the upper and lower chambers were preprocessed with 100 µg mL^−1^ Tf solution to saturate the Tf receptors, and then the cells in the lower chamber were collected for subsequent analysis to compare the effect of Tf preprocessing and untreatment.

### The Evaluation of Photothermal Performance

Pg, EV‐H‐T, Pg@EV‐H‐T solution in tubes were treated with 0.33 W cm^−2^ 808 nm for 10 min. The temperature change was recorded every 1 min. Infrared thermal imaging of Pg, EV‐H‐T, and Pg@EV‐H‐T solution and mice treated with those solutions before or after 808 nm laser irradiation were recorded. The live/dead cell assay was used to examine the laser‐induced cell death according to the corresponding scheme of a test kit.

### Antioxidation Experiment

Confocal microscopy was used to detect Rosup preprocessed BV2 cells. DCFH‐DA dye was used to label the cellular ROS content. Meanwhile, HO‐1 concentration, hemin, Fe^3+^, and Fe^2+^ levels in BV2 cells were measured.

### Targeting Behavior and Safety of the Customized Bacteria In Vivo

To demonstrate the Pg@EV‐H‐T using intranasal administration can improve the targeting behavior into the glioma, intraperitoneal injections, intravenous injections, and intranasal administration were carried out. Here, 100 µL DiR labeled Pg@EV‐H‐T or 10 µL Pg@EV‐H‐T (each containing 20 µg EV, 7.29 µm hemin, 0.25 µm transferrin, and 5 µm DiR) were employed. Intraperitoneal injections and intravenous injection used 100 µL while intranasal administration used 10 µL. Images were obtained in the prone position at 3 h. To further verify the distribution of Pg@EV‐H‐T through intranasal administration, mice were euthanized and their main organs were excised at time points of 1, 3, 6, 12, and 24 h. The fluorescence images were captured and analyzed using in vivo imaging system with a DiR filter channel.

### Biosafety Studies

The blood samples from Pg@EV‐H‐T or PBS‐treated mice were collected and performed a complete blood panel analysis and blood biochemical analysis. After laser treatment, the main organs were made into single cell suspension, plated evenly on blood agar to culture for another 72 h to see if there still had bacteria left after photothermal treatment. Meanwhile, major organs were also sliced and stained with H&E dye. In addition, behavioral tests, including open field and rotarod, were used to analyze the behavioral differences among normal mice, glioma‐bearing mice, and glioma‐bearing mice with Pg@EV‐H‐T treatment.

### Anti‐Tumor Assessment

The day of tumor implantation using a stereotactic fixation device was defined as day 0. After 7 days of U87‐Luc or GL261‐Luc implantation (2 × 10^5^ cells per mouse), the mice were treated with PBS, immunotherapy (anti‐PD‐1), photothermal therapy (Pg@EV‐H‐T+808 nm laser), and photothermal therapy & immunotherapy (anti‐PD‐1+Pg@EV‐H‐T+808 nm laser). The treatment was repeated one time per three days for a total of four times. The tumor size was monitored through relative bioluminescence intensity using the small animal imaging system. On day 21, T2‐weighted magnetic resonance imaging was scanned for groups. At the same time, the brain, heart, liver, spleen, lung, and kidney were separated, fixed, and stained with H&E. The brains were also subjected to caspase3, cleaved caspase3, and other stainings. The survival curves were drawn during the period of 60 days.

### The Analysis of Immune Microenvironment

Brains that contain tumors were extracted at day 21, weighed, and added 500 µL PBS for further lysing into cell populations. The cells were resuspended in an antibody stain buffer. APC‐CD11b, FITC‐CD206, Percp/Cy5.5‐CD80, and PE‐CD86 were used as macrophage surface markers. FITC‐CD3, PE‐CD4, and Percp/Cy5.5‐CD8 were used as T‐cell surface markers. The cell‐free supernatants were used for IL‐4 (M2 marker) and IL‐1 (M1 marker) ELISA studies. Besides, total RNA isolation from brain cells was used for reverse transcription. The resulting cDNA was then used for quantified mRNA expression level. GAPDH was the internal reference. iNOS and MCP‐1 were the M2 markers. Arg1 and Ym1 were the M1 markers.

The sequences used were as follows:

GAPDH,

forward: TCGTCCCGTAGACAAAATGG,

reverse: TTGAGGTCAATGAAGGGGTC;

iNOS,

forward: CAGCTGGGCTGTACAAACCTT,

reverse: CATTGGAAGTGAAGCGTTTCG;

MCP‐1,

forward:CCAGCACCAGCACCAGCCAA,

reverse:TGGATGCTCCAGCCGGCAAC;

Arg1,

forward: CTCCAAGCCAAAGTCCTTAGAG,

reverse: AGGAGCTGTCATTAGGGACATC;

Ym1,

forward: CAGGTCTGGCAATTCTTCTGAA,

reverse: GTCT TGCTCATGTGTGTAAGTGA.

### Statistical Analysis

Statistical preprocess were finished with Microsoft Excel (e.g., transformation, normalization, evaluation of outliers). All results were reported as means ± SD and represented a minimum of six independent experiments. Statistical significance between groups (*p < 0.05, **p < 0.01, and ***p < 0.001) was demonstrated by t‐tests or ANOVA. GraphPad Prism 8 software was used to analyze the data.

## Conflict of Interest

The authors declare no conflict of interest.

## Supporting information

Supporting Information

## Data Availability

The data that support the findings of this study are available from the corresponding author upon reasonable request.
