# Supplementary material for: Bacteria Synergized with PD‐1 Blockade Enhance Positive Feedback Loop of Cancer Cells‐M1 Macrophages‐T Cells in Glioma
Source: Adv Sci (Weinh). 2024 Mar 23;11(20):2308124. doi: 10.1002/advs.202308124 (PMC11132069; doi:10.1002/advs.202308124)
Supplement: Supplementary file 1 — Supporting Information [file ADVS-11-2308124-s001.pdf]

ADVANCED SCIENCE
Open Access

Supporting Information

for *Adv. Sci.*, DOI 10.1002/advs.202308124

Bacteria Synergized with PD-1 Blockade Enhance Positive Feedback Loop of Cancer Cells-M1 Macrophages-T Cells in Glioma

Qi Chen, Yuyi Zheng, Xiaojie Chen, Yuan Xing, Jiajie Zhang, Xinyi Yan, Qi Zhang, Di Wu* and Zhong Chen**

Supporting Information

Bacteria synergized with PD-1 blockade enhance positive feedback loop of cancer cells-M1 macrophages-T cells in glioma

Qi Chen*, Yuyi Zheng, Xiaojie Chen, Yuan Xing, Jiajie Zhang, Xinyi Yan, Qi Zhang, Di Wu*, Zhong Chen*

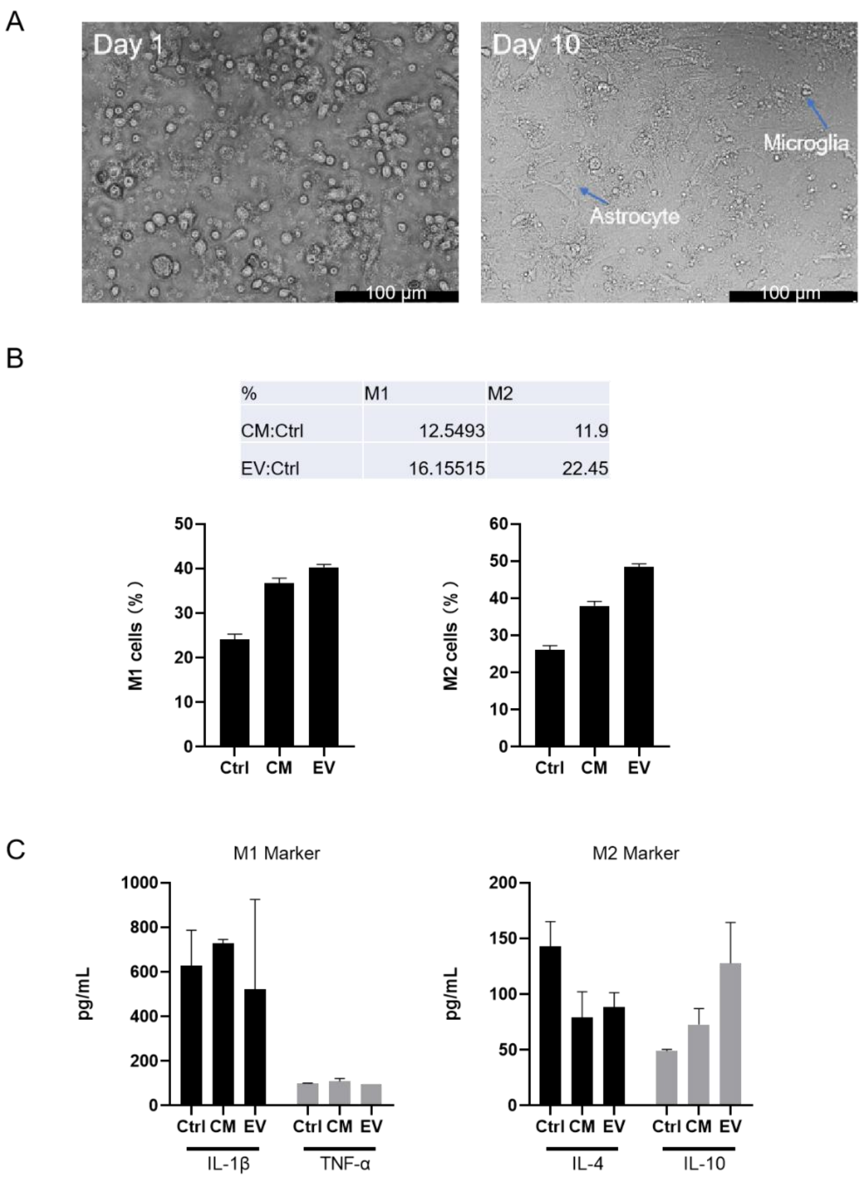

Figure S1. A) The culturable state of primary microglias at day 1 and 10. At day 1, the cells were extracted from the brains of fetal mice and cultured on a T25 flask. At day 10, the cell differentiation was observed. B) Cell membranes (CM) or extracellular vesicles (EV) induced changes in the proportion of microglial cell types (simply divided into M1 and M2) when compared to control (Ctrl) group. C) Expression levels of IL-1β. TNF-α, IL-4, and IL-10 in the primary microglias culture supernatant were measured by ELISA 24 h after the stimulation of CM or EV.

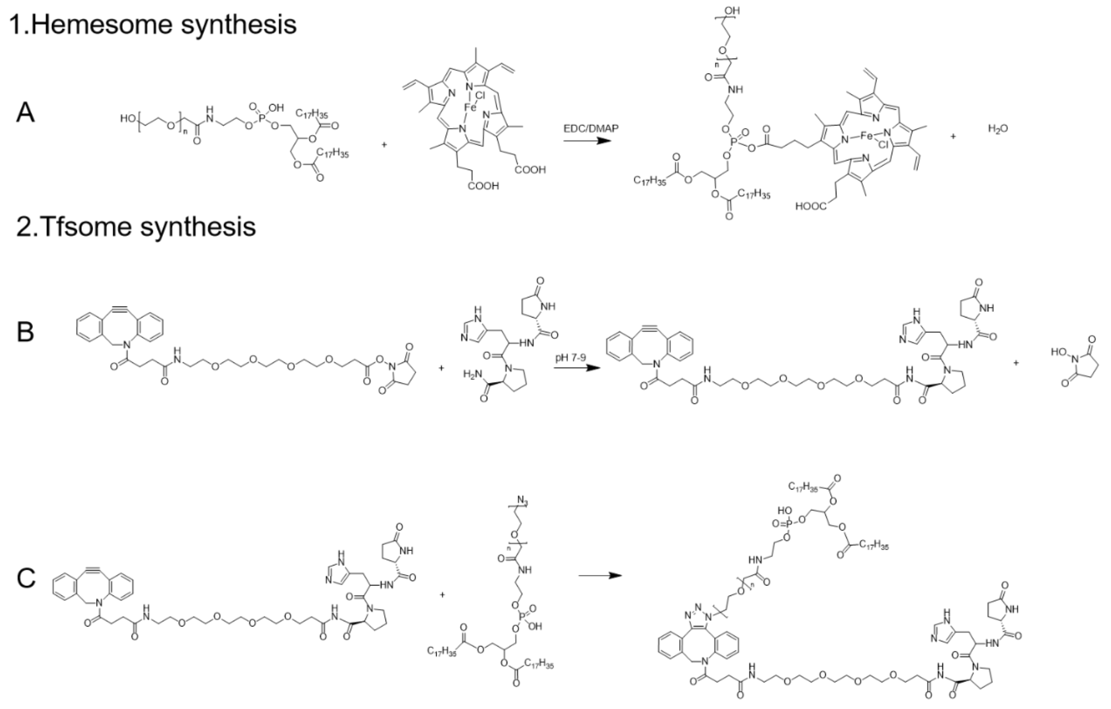

Figure S2. A) Schematic illustration for hemsome systhesis. B) and C) Schematic illustration for Tfsome synthesis.

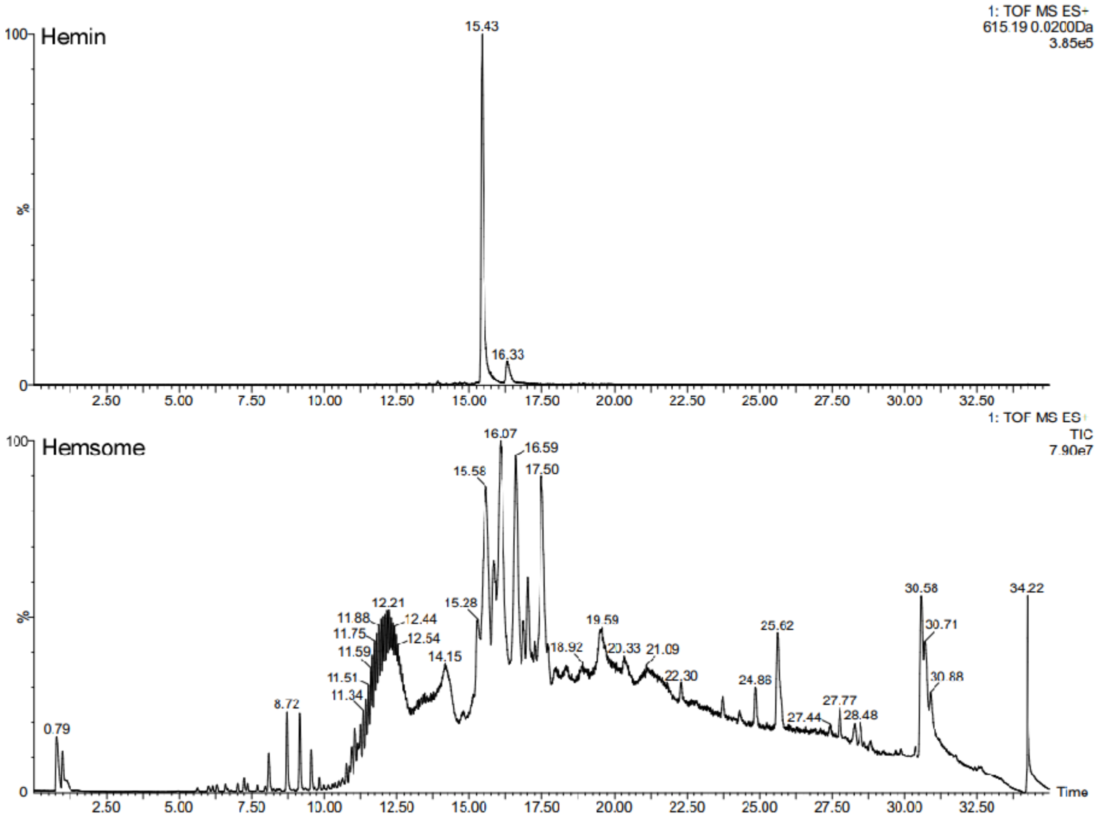

Figure S3. The LC-MS result indicates Hemsomes were successfully obtained.

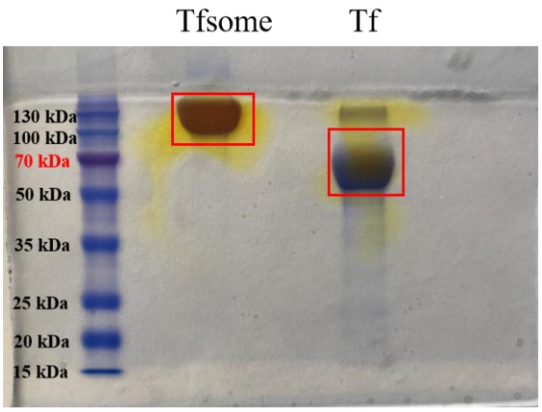

Figure S4. SDS-PAGE&PEG-color reaction of Tfsome and Tf to demonstrate Tfsomes were successfully obtained.

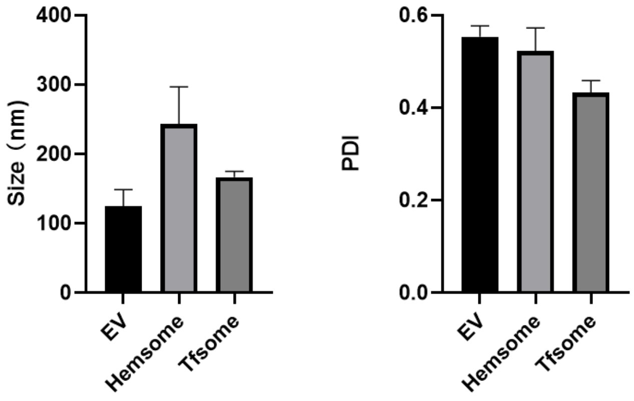

Figure S5. Size and Polydispersity index (PDI) analysis of EV, Hemsome and Tfsome.

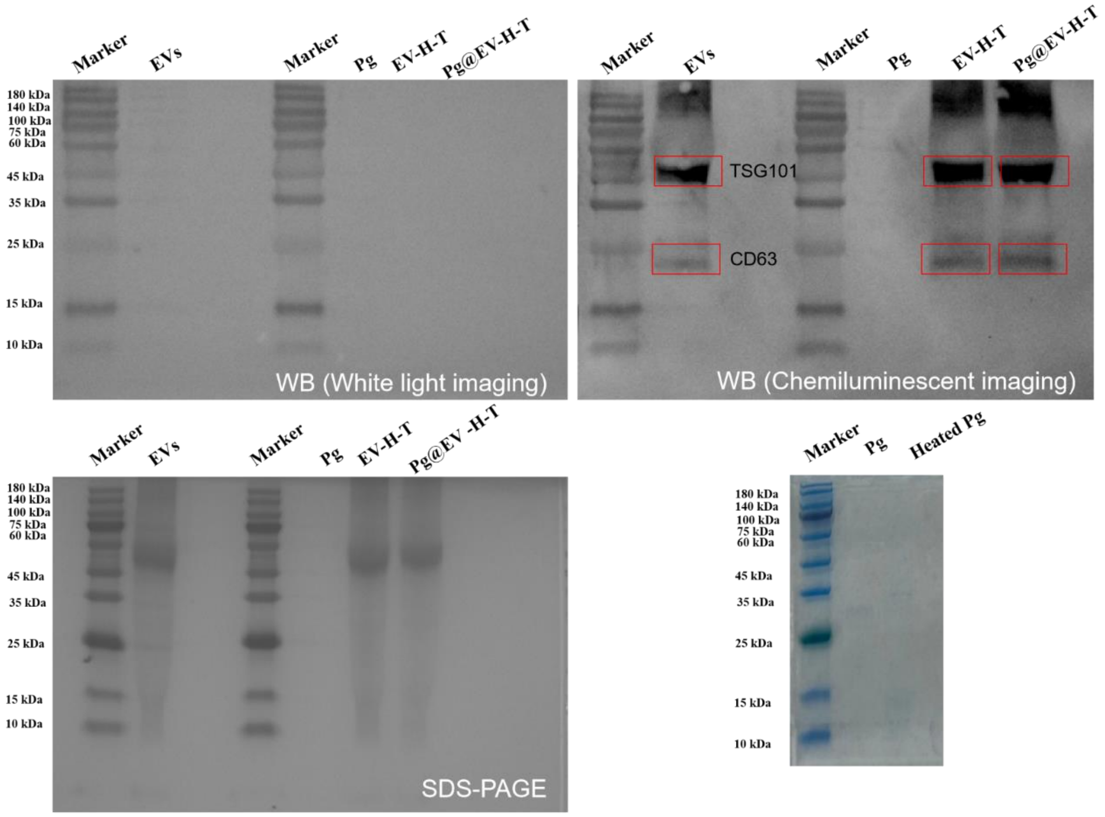

Figure S6. Western blot and SDS-PAGE results show components from EVs could be reserved on Pg@EV-H-T.

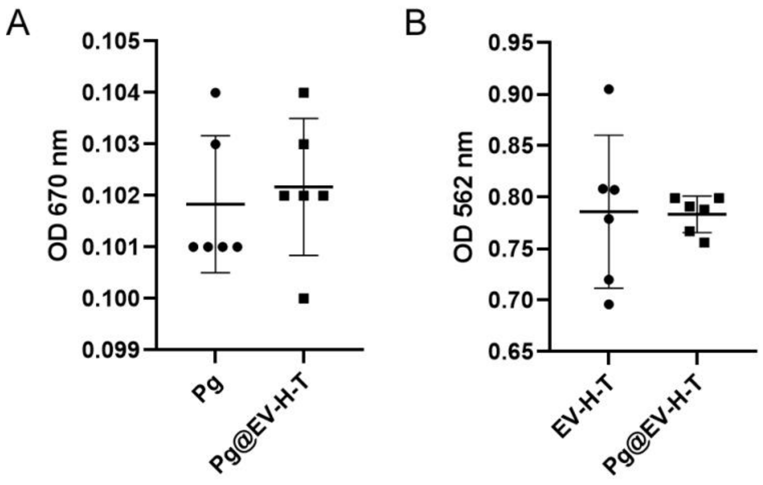

Figure S7. (A) Quantify the bacteria concentration of Pg and Pg@EV-H-T. (B) Quantify the outer protein concentration of EV-H-T and Pg@EV-H-T.

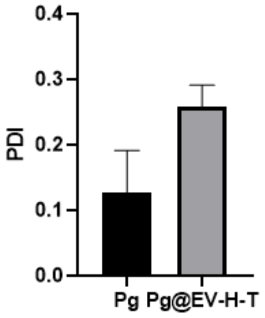

Figure S8. PDI analysis of Pg and Pg@EV-H-T.

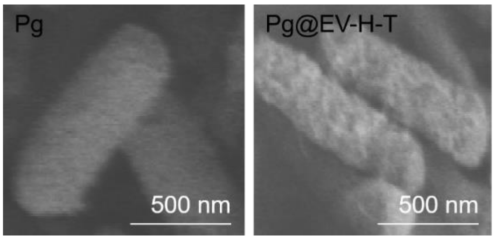

Figure S9. SEM images of Pg and Pg@EV-H-T. Scale bar=500 nm.

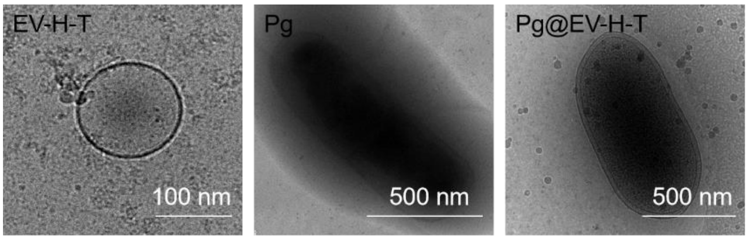

Figure S10. Cryo-Tem images of EV-H-T, Pg and Pg@EV-H-T. Scale bar=500 nm.

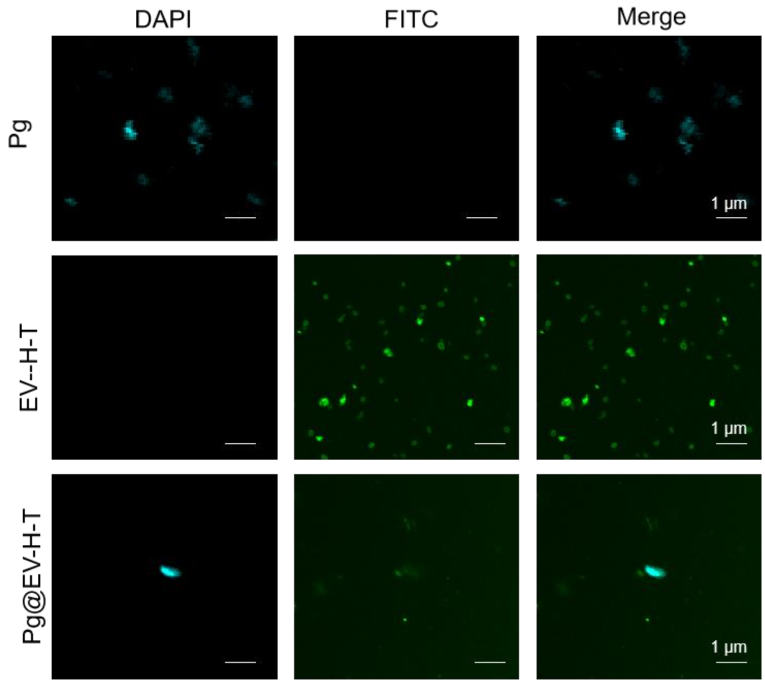

Figure S11. Confocal microscopy images of Pg, EV-H-T and Pg@EV-H-T. Pg was labeled with DAPI and EV-H-T was labeled with FITC. Pg@EV-H-T had signals from two channel. Scale bar=1 μm.

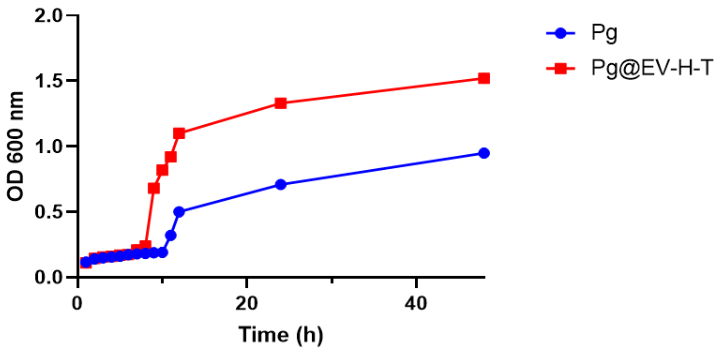

Figure S12. Quantify bacterial growth of Pg and Pg@EV-H-T.

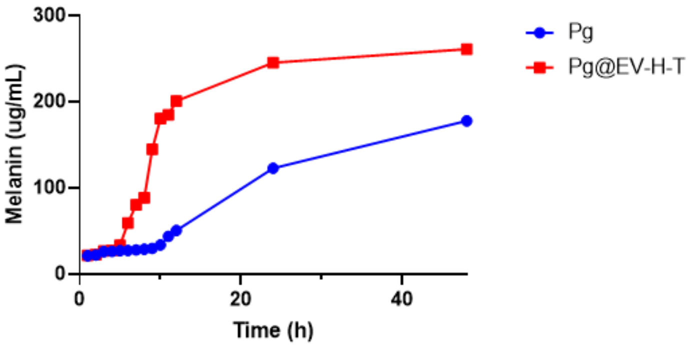

Figure S13. Quantify the production of melanin from Pg and Pg@EV-H-T.

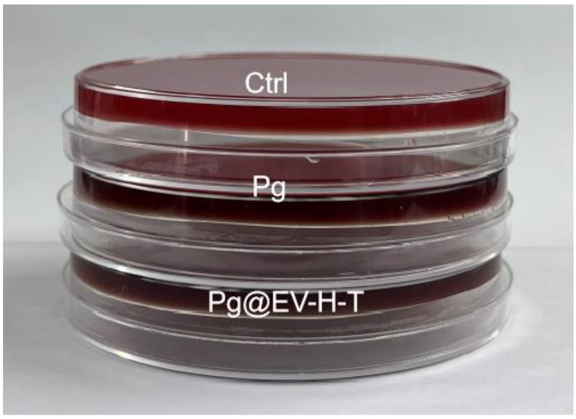

Figure S14. The imges of Pg and Pg@EV-H-T cultured blood agar showing the presence of melanin.

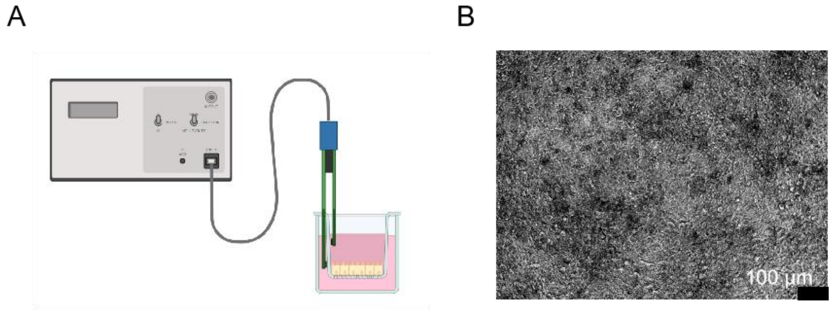

Figure S15. A) Schematic of the measure of the transepithelial electrical resistance (TEER) from transwells using an epithelial voltmeter. B) The image of confluent bEnd.3 under microscope. The scale bar=100 μm.

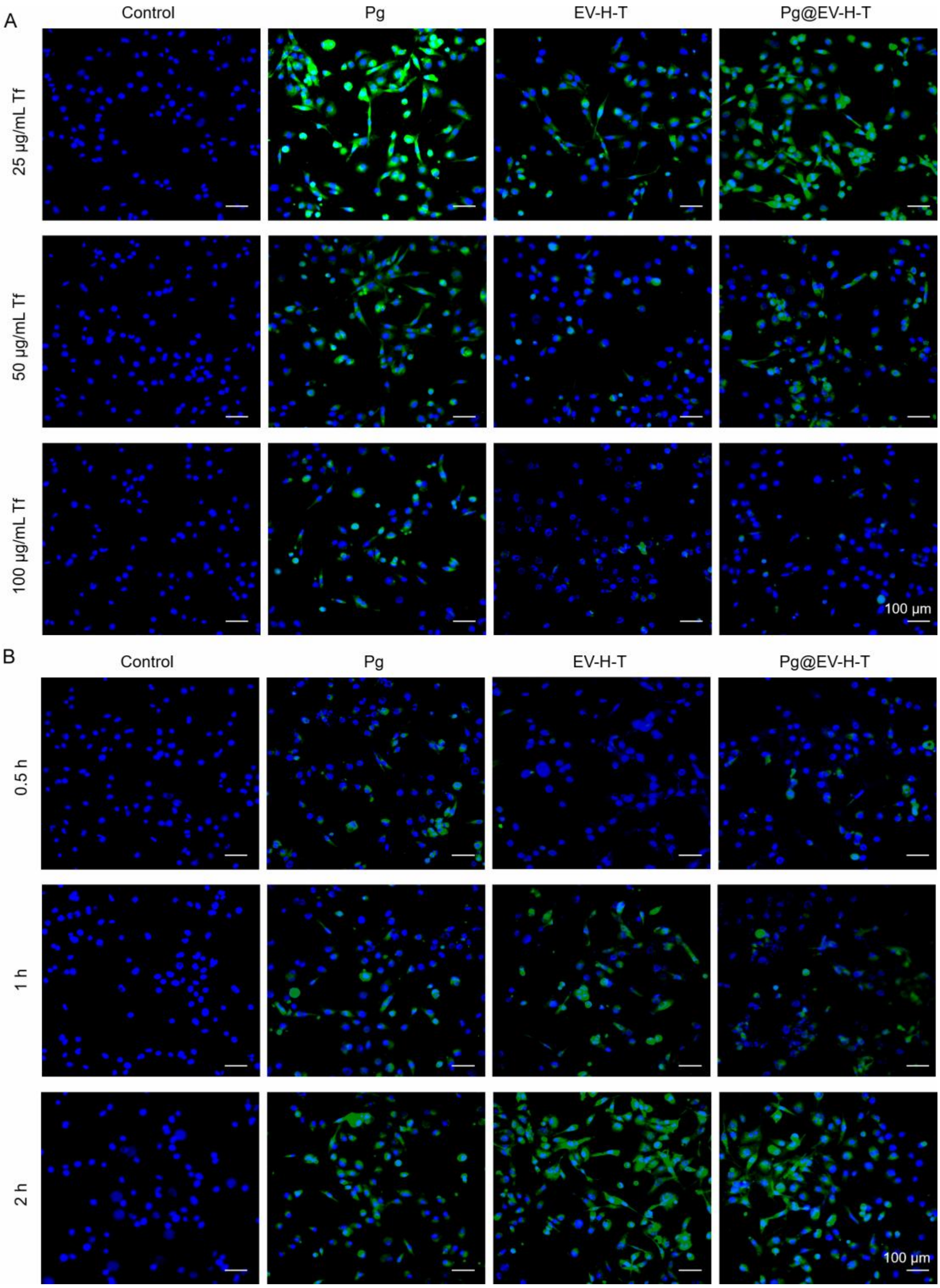

Figure S16. (A) Confocal images of cellular uptake using different concentrations of transferrin to block transferrin receptors, followed by coculture with FITC labeled Pg, EV-H-T, and Pg@ EV-H-T for 0.5 h. (B) Confocal images of cellular uptake using 100 μg/mL transferrin to block transferrin receptors, followed by coculture with FITC labeled Pg, EV-H-T, and Pg@ EV-H-T for 0.5 h, 1 h or 2 h.

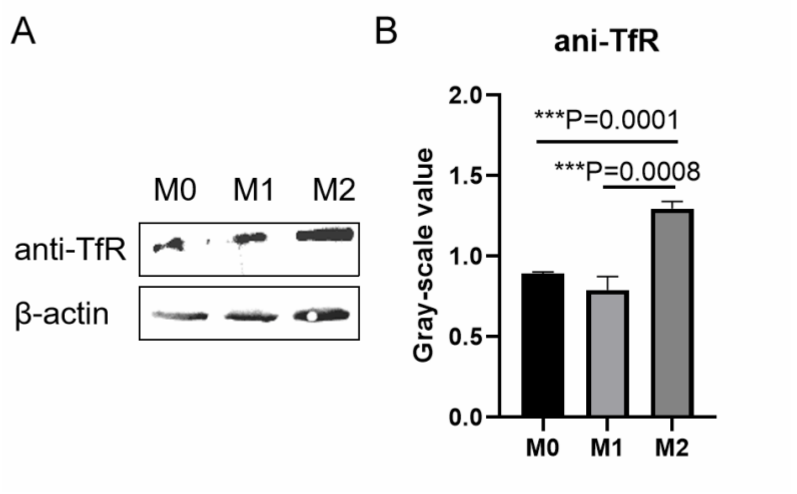

Figure S17. (A) Western blot analysis of levels of TfR in M0, M1 and M2 cell samples. (B) Quantification of the protein abundance of TfR.

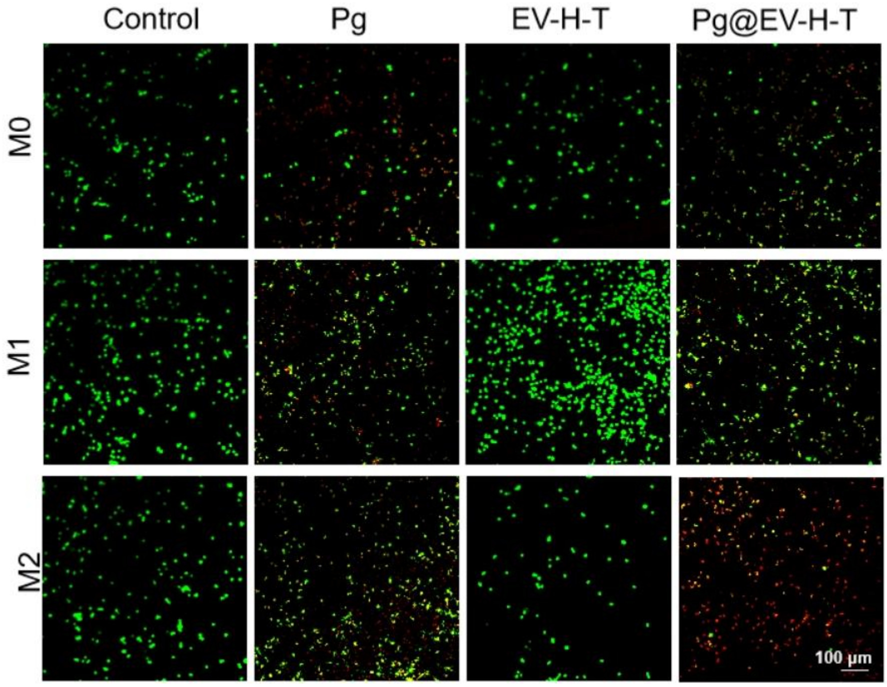

Figure S18. Calcein-AM/PI staining assay of M0, M1 and M2 cells treated with Pg, EV-H-T or Pg@EV-H-T and irradiated with 808 nm laser (0.33 W/cm2, 10 min). Scale bar=100 μm.

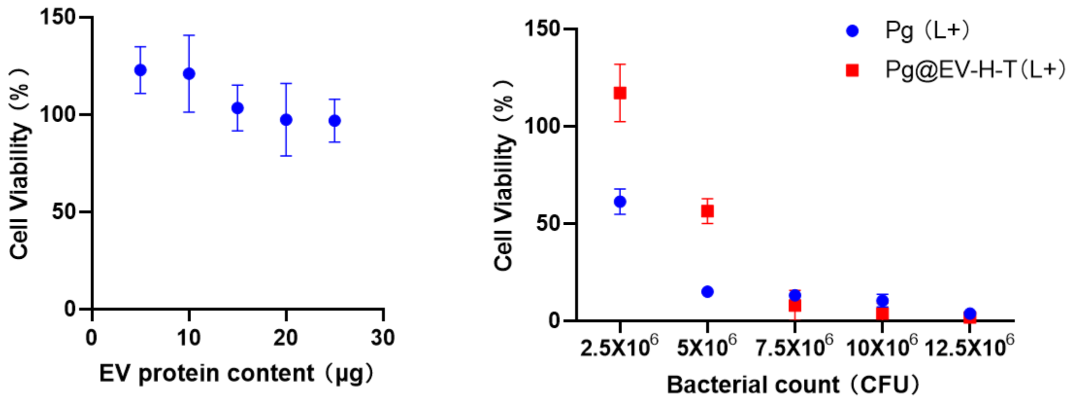

Figure S19. In vitro anti-glioma effect of EV-H-T, Pg and Pg@EV-H-T with the laser treatment. SD=6.

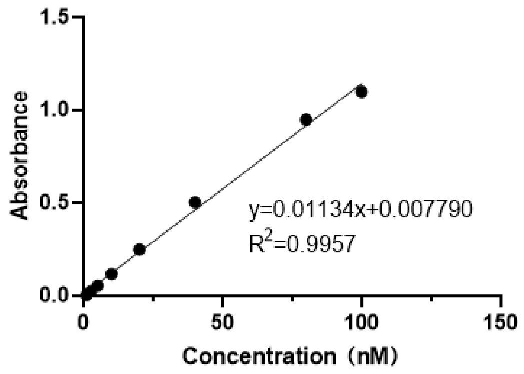

Figure S20. UV absorption standard curve of hemin.

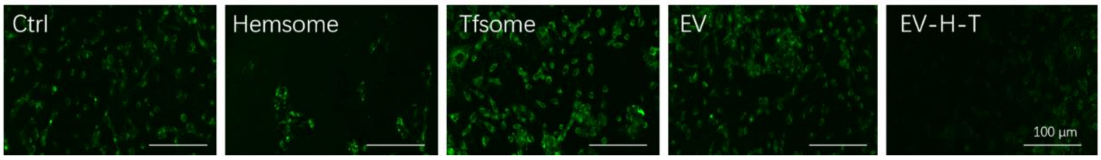

Figure S21. Fluorescent images of ROS levels in BV2 cells with Hemsome, Tfsome, EV and EV-H-T treatments. The cells were stained by green fluorescence DCFH-DA. Scale bar=100 μm.

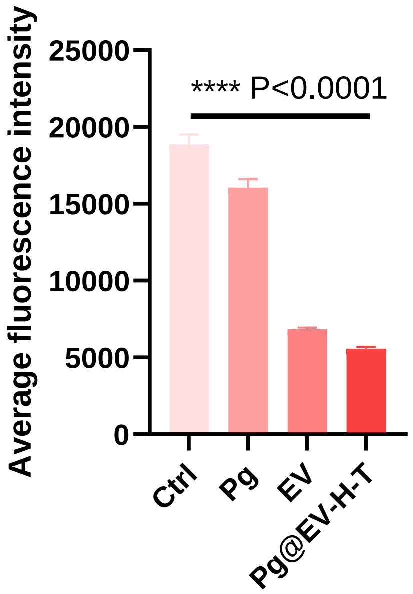

Figure S22. (A) Average DCFH-DA fluorescence intensity in BV2 cells.

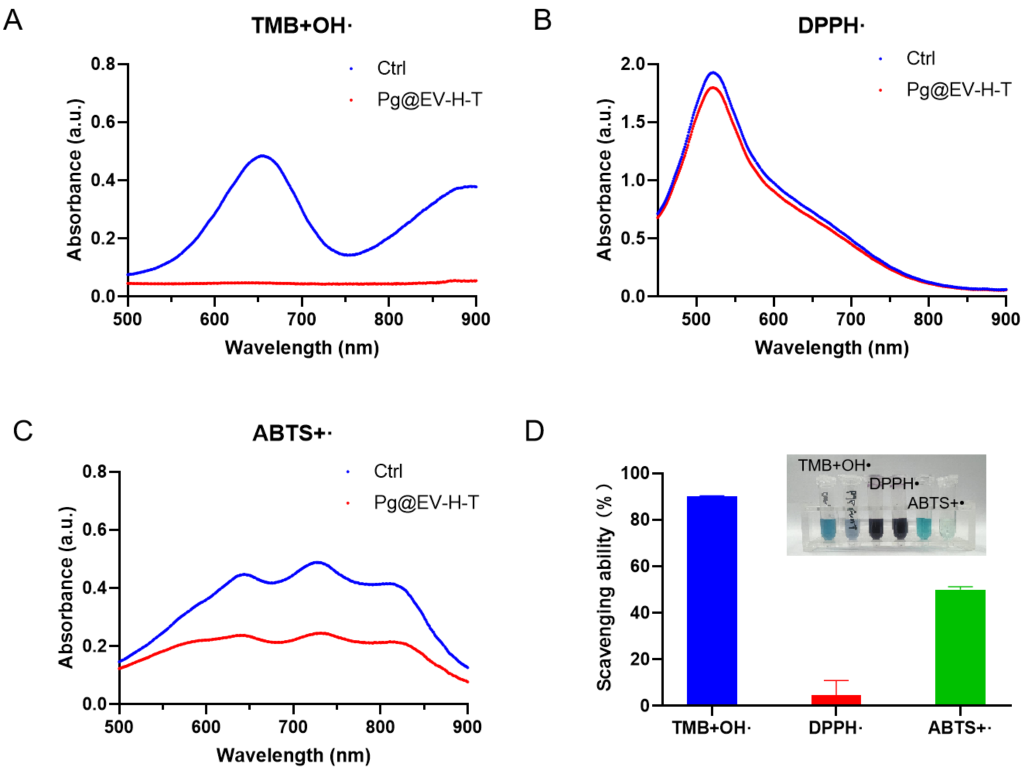

Figure S23. A) Absorbance spectra of TMB+OH•radicals after incubation with Pg@EV-H-T. B) Absorbance spectra of DPPH•radicals after incubation with Pg@EV-H-T. C) Absorbance spectra of ABTS+•radicals after incubation with Pg@EV-H-T. D) Photograph and scavenging effects of Pg@EV-H-T.

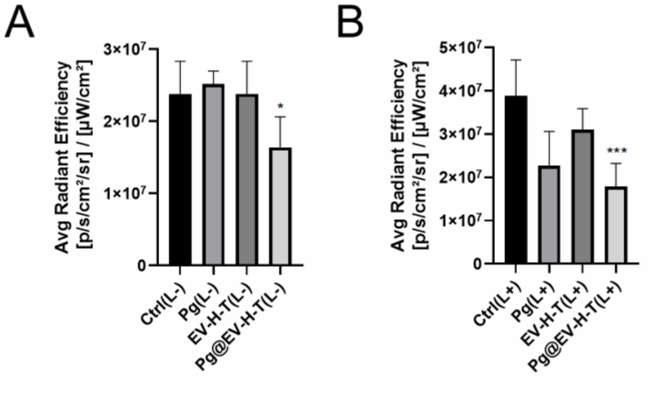

Figure S24. (A) Quantitative analysis of ROS by DCFH-DA in M1 cells after incubation with Pg, EV-H-T and Pg@EV-H-T. (B) Quantitative analysis of ROS by DCFH-DA in M1 cells after incubation with Pg, EV-H-T and Pg@EV-H-T and treatment with 808 nm laser.

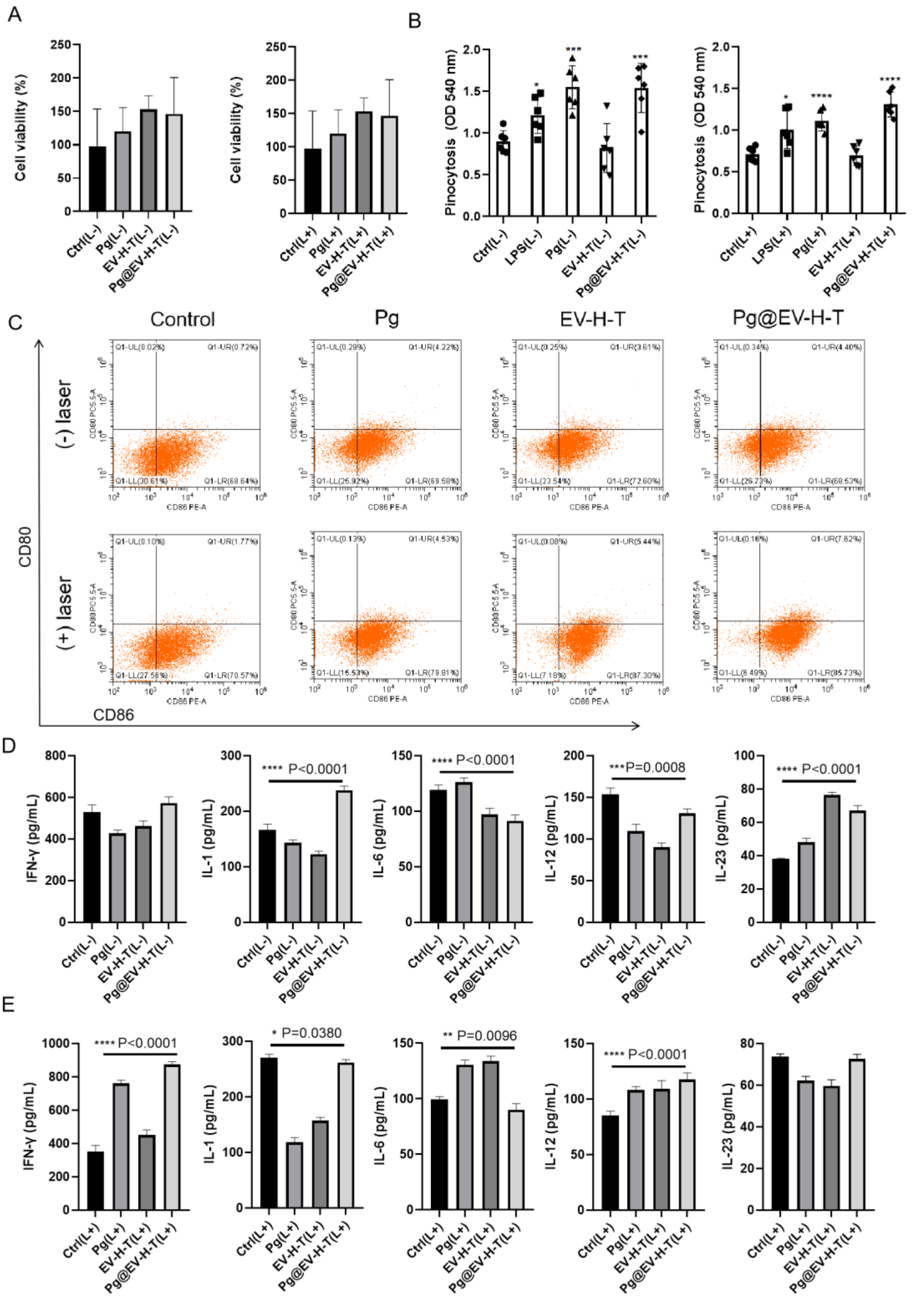

Figure S25. (A) CCK-8 assay was used to detect M1 cells viability after incubation with Pg, EV -H-T and Pg@EV-H-T and then treatment with or without 808 nm laser. (B) M1 pinocytosis after incubation with Pg, EV-H-T and Pg@EV-H-T and then treatment with or without 808 nm laser. (C) Expression of CD80 and CD86 on M1

macrophages after incubation with Pg, EV-H-T and Pg@EV-H-T and then treatment with or without 808 nm laser. (D) The ability of M1 macrophages to secrete cytokines after incubation with Pg, EV-H-T and Pg@EV-H-T. (E) The ability of M1 macrophages to secrete cytokines after incubation with Pg, EV-H-T and Pg@EV-H-T and treatment with laser.

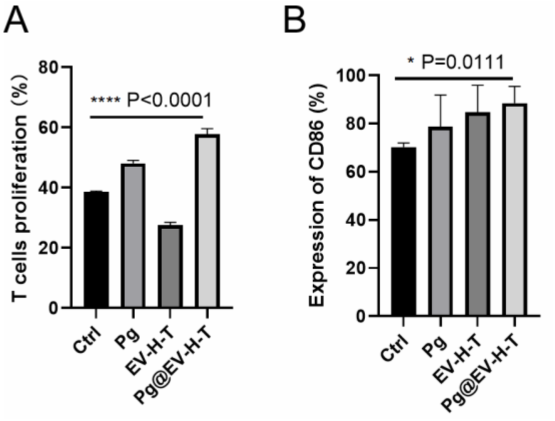

Figure S26. (A) Quantification of T-cell proliferation in the transwell system. (B) Quantification of CD86 expression on M1 cells.

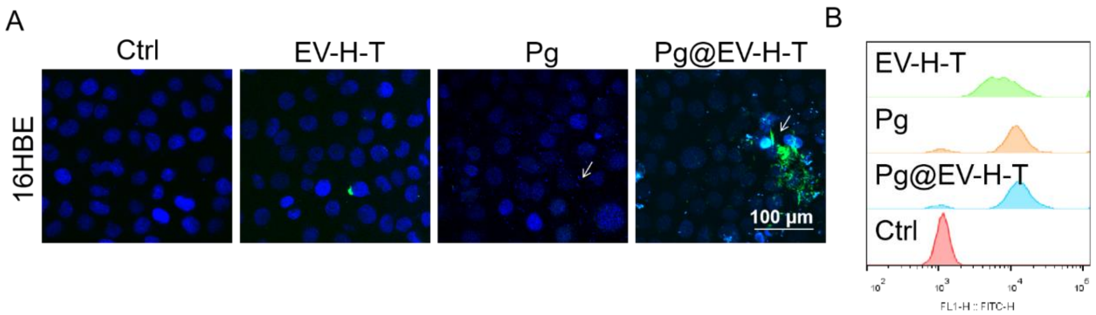

Figure S27. A) Confocal images of 16HBE cells after incubation with EV-H-T, Pg and Pg@EV-H-T. EV-H-T, Pg and Pg@EV-H-T were stained by green fluorescence FITC. The cells were stained by blue fluorescence DAPI. Scale bar=100 μm. B) Flow cytometric analysis of 16HBE cells following incubation with different groups.

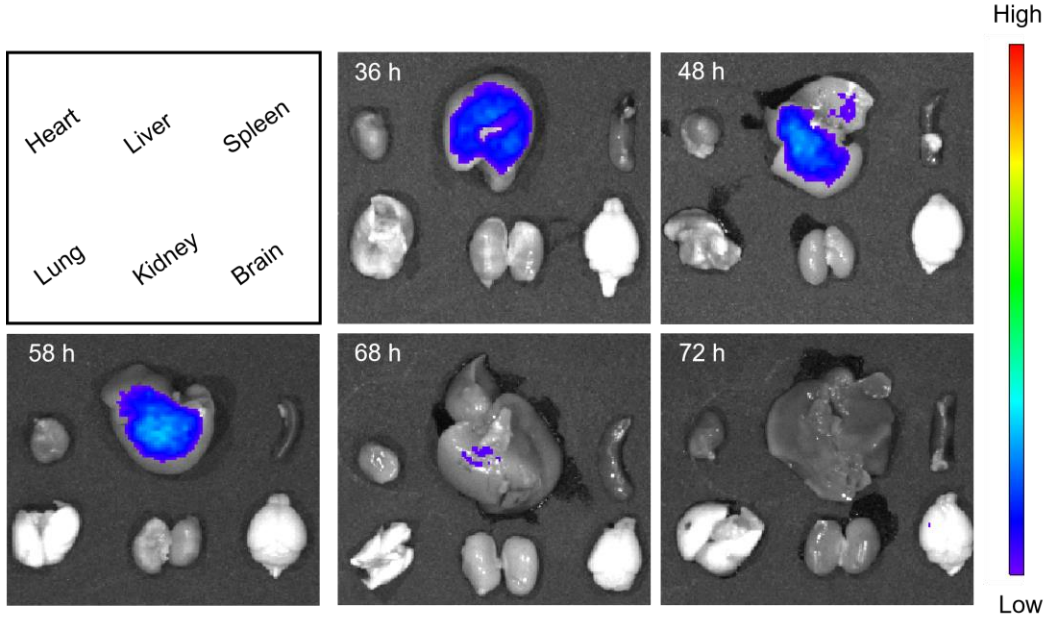

Figure S28. Fluorescence images of major tissues in 36 h to 72 h of U87-bearing mice indicate the decomposition of Pg@EV-H-T in the body.

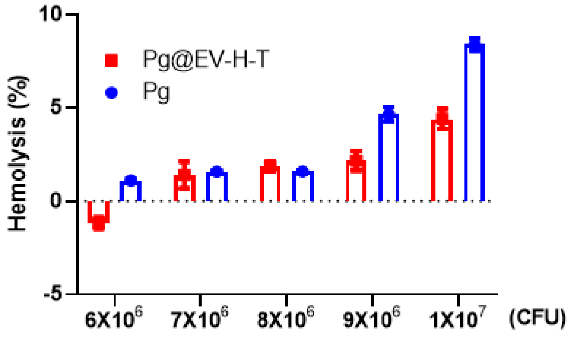

Figure S29. Hemolysis of RBCs incubated with Pg or Pg@EV-H-T at different CFUs ranging from 6X 10^6 CFU to 1X10^7 CFU for 3 h.

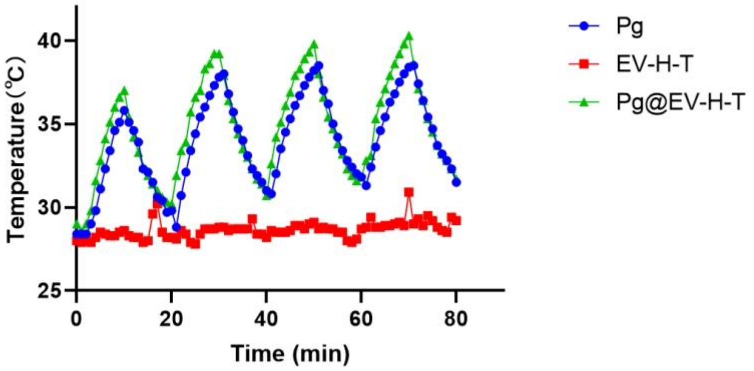

Figure S30. Heating of the Pg, EV-H-T and Pg@EV-H-T in mice for four laser on/off cycles.

A

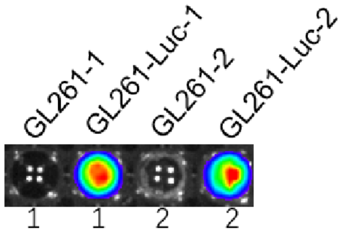

B

Cells ($2x10^5$)	Total Flux [p/s]-1	Total Flux [p/s]-2	Average	SD	Average total flux [p/s]/Cells
GL261	2.24E+05	2.00E+05	2.12E+05	1.71E+04	1.06
GL261-Luc	1.10E+08	9.79E+07	1.04E+08	8.58E+06	519.68

Figure S31. A) Bioluminescence images for ensuring the Lentiviral transduction of GL261 tumor cells with firefly luciferase. B) Quantification of bioluminescence from GL261-Luc cells through measuring total flux (p/s) and dividing them to each cell.

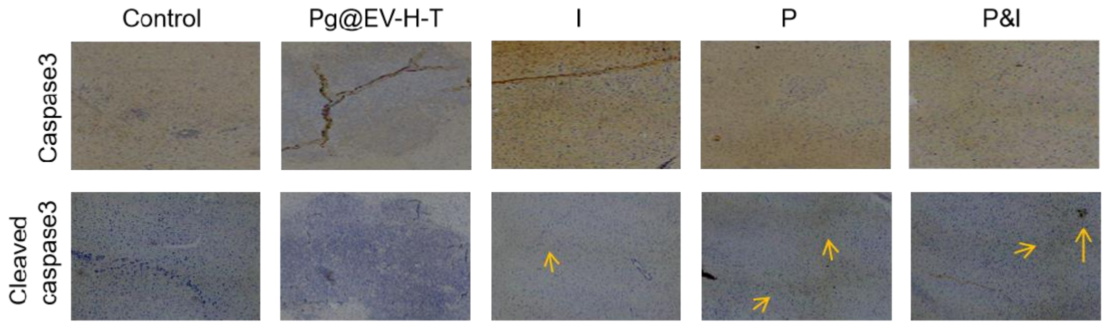

Figure S32. Immunohistochemistry of caspase3, cleaved caspase3, and TUNEL on the brain slides from GL261 glioma models with different treatments. Yellow arrows point to positive expression.

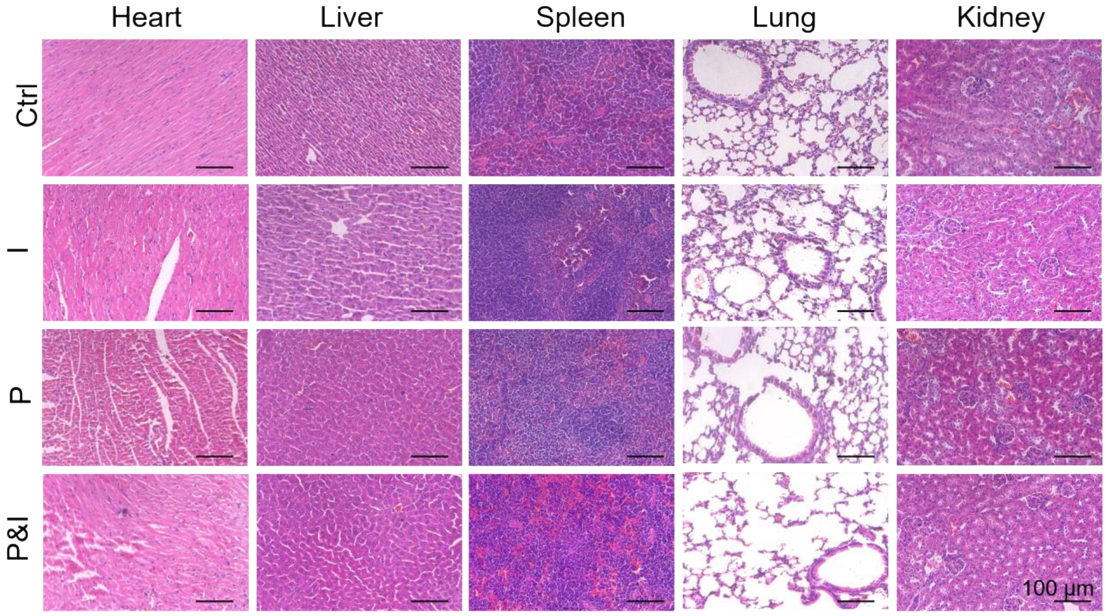

Figure S33. H&E staining assays on heart, liver, spleen, lung and kidney of mice from Ctrl, I, P and P&I groups. Scale bar=100 μm.

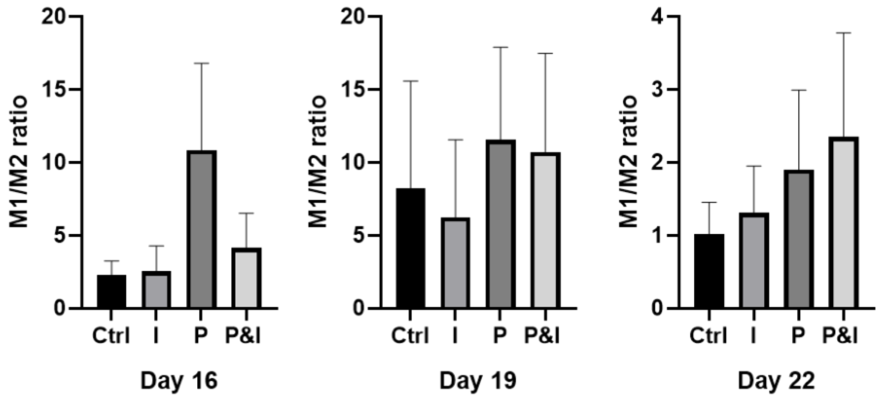

Figure R34. The M1/M2 ratio in the brains after different treatments at Day 16 v. Day 19 v. Day 22.

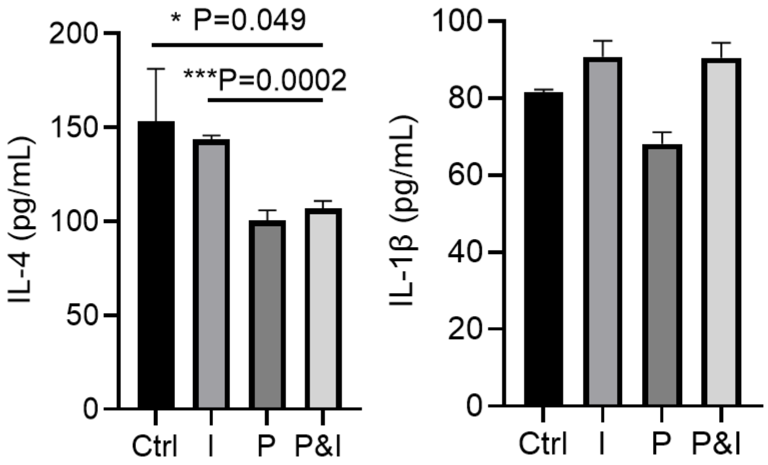

Figure S35. Expression levels of IL-4 and IL-1β in the brain.
